# Preexisting immunity restricts mucosal antibody recognition of SARS-CoV-2 and Fc profiles during breakthrough infections

**DOI:** 10.1172/jci.insight.172470

**Published:** 2023-09-22

**Authors:** Kevin J. Selva, Pradhipa Ramanathan, Ebene R. Haycroft, Arnold Reynaldi, Deborah Cromer, Chee Wah Tan, Lin-Fa Wang, Bruce D. Wines, P. Mark Hogarth, Laura E. Downie, Samantha K. Davis, Ruth A. Purcell, Helen E. Kent, Jennifer A. Juno, Adam K. Wheatley, Miles P. Davenport, Stephen J. Kent, Amy W. Chung

**Affiliations:** 1 Department of Microbiology and Immunology, Peter Doherty Institute for Infection and Immunity, University of Melbourne, Melbourne, Victoria, Australia.; 2 Kirby Institute, University of New South Wales, Kensington, New South Wales, Australia.; 3 Programme in Emerging Infectious Diseases, Duke-NUS Medical School, Singapore.; 4 Infectious Diseases Translational Research Programme, Department of Microbiology and Immunology, Yong Loo Lin School of Medicine, National University of Singapore, Singapore.; 5 Singhealth Duke-NUS Global Health Institute, Singapore.; 6 Immune Therapies Laboratory, Burnet Institute, Melbourne, Victoria, Australia.; 7 Department of Immunology and Pathology, Central Clinical School, Monash University, Melbourne, Victoria, Australia.; 8 Department of Clinical Pathology, University of Melbourne, Melbourne, Victoria, Australia.; 9 Department of Optometry and Vision Sciences, University of Melbourne, Carlton, Victoria, Australia.; 10 Melbourne Sexual Health Centre and Department of Infectious Diseases, Alfred Hospital and Central Clinical School, Monash University, Melbourne, Victoria, Australia.

**Keywords:** COVID-19, Adaptive immunity

## Abstract

Understanding mucosal antibody responses from SARS-CoV-2 infection and/or vaccination is crucial to develop strategies for longer term immunity, especially against emerging viral variants. We profiled serial paired mucosal and plasma antibodies from COVID-19 vaccinated only vaccinees (vaccinated, uninfected), COVID-19–recovered vaccinees (recovered, vaccinated), and individuals with breakthrough Delta or Omicron BA.2 infections (vaccinated, infected). Saliva from COVID-19–recovered vaccinees displayed improved antibody-neutralizing activity, Fcγ receptor (FcγR) engagement, and IgA levels compared with COVID-19–uninfected vaccinees. Furthermore, repeated mRNA vaccination boosted SARS-CoV-2–specific IgG2 and IgG4 responses in both mucosa biofluids (saliva and tears) and plasma; however, these rises only negatively correlated with FcγR engagement in plasma. IgG and FcγR engagement, but not IgA, responses to breakthrough COVID-19 variants were dampened and narrowed by increased preexisting vaccine-induced immunity against the ancestral strain. Salivary antibodies delayed initiation following breakthrough COVID-19 infection, especially Omicron BA.2, but rose rapidly thereafter. Importantly, salivary antibody FcγR engagements were enhanced following breakthrough infections. Our data highlight how preexisting immunity shapes mucosal SARS-CoV-2–specific antibody responses and has implications for long-term protection from COVID-19.

## Graphical abstract

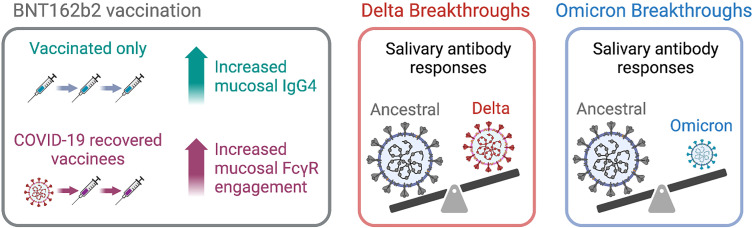

## Introduction

COVID-19 vaccines, and particularly mRNA boosters, elicit SARS-CoV-2–specific neutralizing antibodies systemically and protect against severe disease. However, current intramuscular (IM) COVID-19 vaccination regimes among SARS-CoV-2–uninfected individuals induce limited site-specific neutralizing antibodies at the mucosa — the site of SARS-CoV-2 acquisition (1). This gap in mucosal humoral immunity is thought to contribute to vaccine breakthrough infections (2, 3).

Prior SARS-CoV-2 infection primes improved mucosal antibody responses elicited by subsequent vaccinations (2, 4–6). Previous studies have largely focused on the neutralizing potential of mucosal antibody isotypes IgG and IgA, with little known about mucosal antibody subclass responses (IgG1-4, IgA1-2), each of which have unique profiles and functions. Furthermore, while the retention of antibody-mediated functional responses, despite the waning of neutralization, has been demonstrated in the blood, its potential at the mucosal surface remains understudied (7).

Preexisting vaccine-induced immunity may also modulate immune responses during breakthrough infections. Breakthrough infections with the more divergent Omicron BA.1 strain is associated with a more modest recall of SARS-CoV-2 spike immunity as compared with Delta breakthroughs (8). Immunological imprinting from repeated vaccinations with the ancestral spike may hamper the development of robust systemic humoral responses specifically against Omicron during breakthrough infections (9–11). Unfortunately, most studies have only focused on systemic antibodies, and the impact of prior ancestral strain vaccination on mucosal antibodies following breakthrough infection is unclear.

Herein, we compare COVID-19–recovered (recovered, vaccinated) and COVID-19 vaccinated only vaccinees (vaccinated, uninfected), demonstrating that recovered individuals elicit stronger mucosal antibodies following vaccination, with higher capacity to engage Fc receptors. Furthermore, using a series of paired mucosal and plasma samples collected very early following Delta and Omicron BA.2 breakthrough infections (vaccinated then infected), we demonstrate that preexisting immunity also differentially impacts mucosal immunity.

## Results

### Mucosal IgG4 is elevated after third mRNA vaccine dose.

Individuals infected with COVID-19 prior to SARS-CoV-2 vaccinations (COVID-19–recovered vaccinees) elicit stronger systemic total IgG and neutralization responses than those induced only by SARS-CoV-2 vaccination alone (2, 4–6). Furthermore, prior mucosal exposure due to SARS-CoV-2 infection primes improved mucosal total IgG and IgA responses resulting from subsequent IM COVID-19 vaccinations (2, 4–6). However, few studies have delved into the antibody subclass expression, as well as the capacity for antibody-mediated Fc receptor (FcR) engagement, particularly at the mucosa, within such vaccinees.

**Figure 1:**
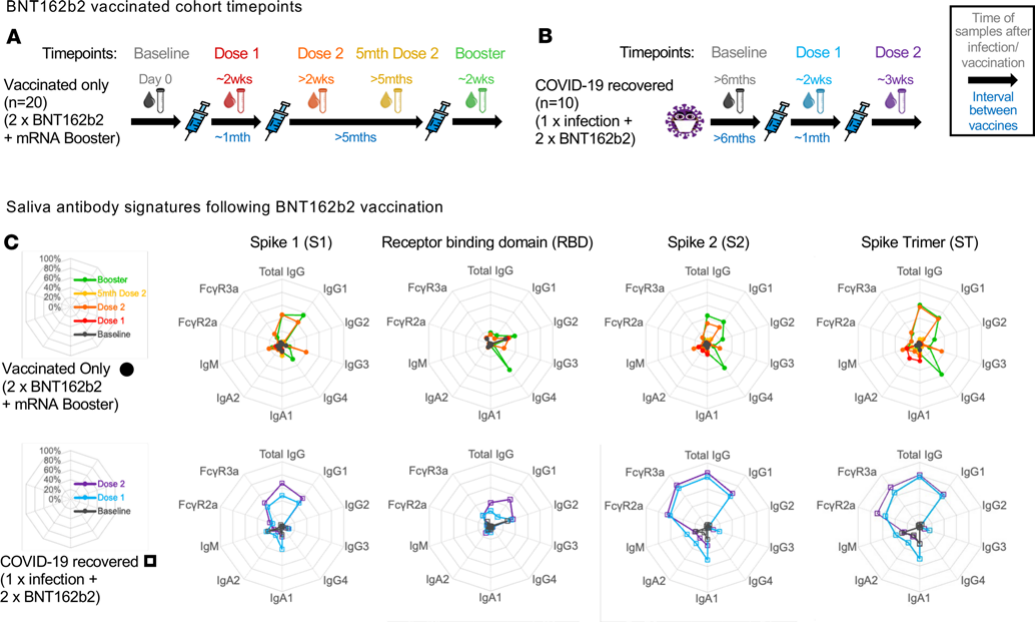
Salivary antibodies from COVID-19–recovered individuals show stronger IgA and FcγR engagement responses after COVID-19 mRNA vaccination. Paired saliva and plasma samples were collected before and after mRNA vaccination from vaccinated only (*n* = 20) (**A**) and COVID-19–recovered (*n* = 10) (**B**) individuals at the indicated time points. Saliva antibody isotype and subclass responses from both cohorts against the various SARS-CoV-2 spike antigens were compiled into respective radar plots (**C**). The individual median antibody isotype/subclass response for each spike antigen was transformed into percentages using the antigen-specific MFI from the 98th percentile for that detector (98th percentile was chosen to minimize the impact of outliers on the data transformation).

To address this, we profiled SARS-CoV-2–specific salivary antibody isotypes, subclasses, and capacity for FcR engagement from both COVID-19 vaccinated only vaccinees receiving up to 3 mRNA vaccines (vaccinated only; 2 × BNT162b2 + 1 × mRNA booster), and COVID-19–recovered vaccinees receiving up to 2 mRNA vaccines (COVID-19 recovered; 1 × prior COVID infection + 2 × BNT162b2) (Figure 1, A–C; cohort information described in Supplemental Figure 1A and Supplemental Table 1; supplemental material available online with this article; https://doi.org/10.1172/jci.insight.172470DS1). The multiplex array used contained both ancestral SARS-CoV-2 receptor binding domain (RBD) and spike 1 (S1) to study novel responses made against SARS-CoV-2, as well as ancestral SARS-CoV-2 spike 2 (S2) and whole spike trimer (ST), which detect cross-reactive responses conserved across other coronaviruses (Supplemental Figure 1B) (12).

**Figure 2:**
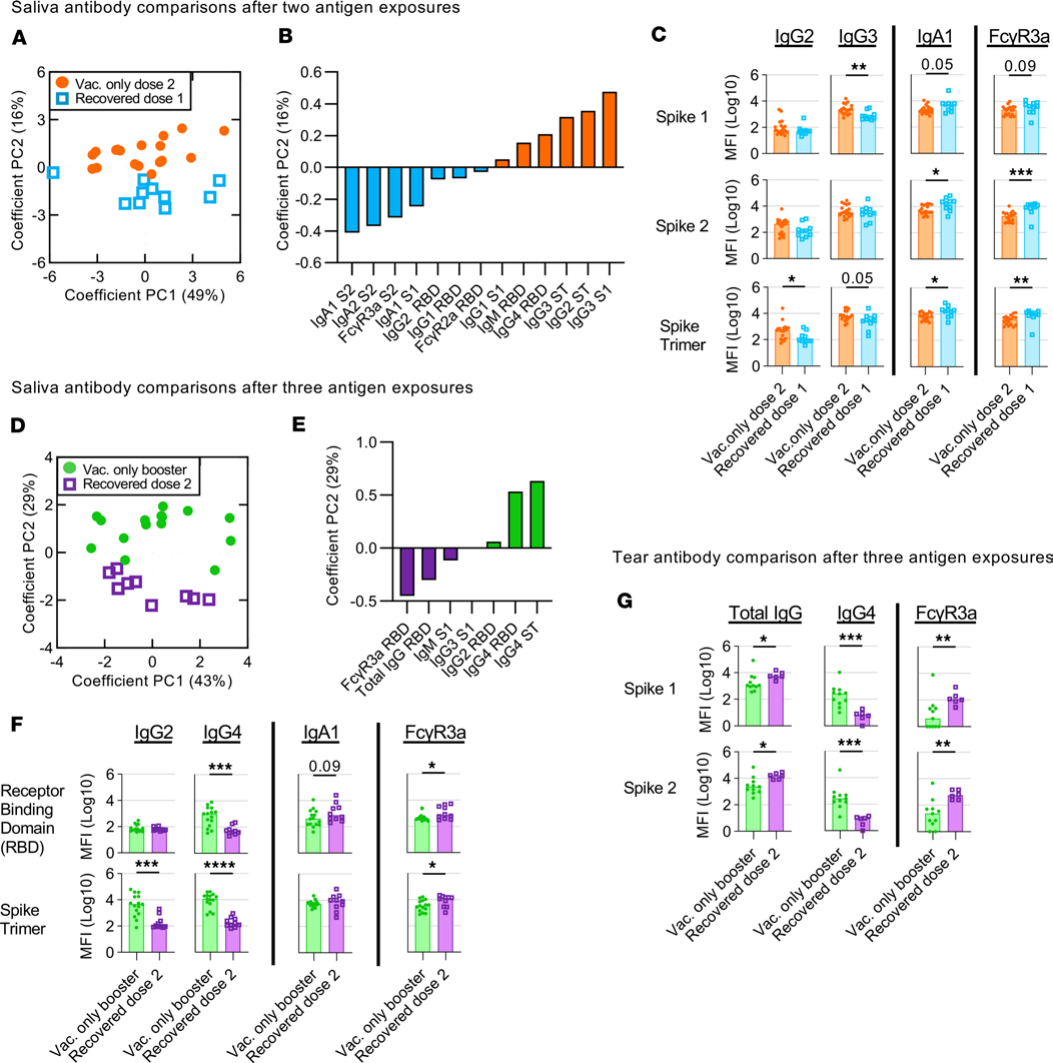
Salivary and tear IgG4 responses are enhanced after the third COVID-19 mRNA vaccine. Principal component analysis (PCA) of all 40 antibody features for vaccinated only (closed circles) (*n* = 20) and COVID-19–recovered (open squares) (*n* = 10) individuals after 2 (**A**) and 3 (**D**) antigen exposures. Loading plots and bar graphs describe the key differences between both cohorts after 2 (**B** and **C**) and 3 (**E** and **F**) antigen exposures. Major tear antibody features after 3 antigen exposures are also illustrated in bar graphs (**G**). Statistical significance was calculated using the 2-tailed Mann-Whitney *U* test and where significant or trending significance, *P* values were reported (*P ≤ 0.05; **P ≤ 0.01; ***P ≤ 0.001; ****P ≤ 0.0001).

As SARS-CoV-2–specific humoral responses improve cumulatively after each antigen exposure (infection or vaccination), we decided it would be fairer to compare responses after an equal number of SARS-CoV-2 exposures (5). After 2 antigen exposures, we detected marked differences in salivary antibody signatures between COVID-19–recovered (1 × prior infection + 1 × BNT162b2) and vaccinated only cohorts (2 × BNT162b2) (Figure 2, A and B). As compared with the vaccinated only cohort, COVID-19–recovered vaccinees developed better salivary IgA responses (Figure 2, B and C, and Supplemental Figure 2A). However, we noted that these salivary IgA responses were biased toward the more conserved spike proteins, S2 and ST, responses (*P* ≤ 0.05) instead of the novel S1 or RBD (Figure 2, B and C, and Supplemental Figure 2A). Importantly, salivary antibodies from COVID-19–recovered vaccinees displayed higher FcγR engagement following 2 antigen exposures (1 × prior infection + 1 × BNT162b2) as compared with the vaccinated only cohort (2 × BNT162b2), also primarily against the conserved antigens S2 and ST (*P* ≤ 0.01) (Figure 2, B and C, and Supplemental Figure 3A). On the other hand, salivary IgG subclass responses in vaccinated only vaccinees (2 × BNT162b2) (Figure 2, B and C, and Supplemental Figure 2A) aligned with that observed in plasma (Supplemental Figure 3, A–D), with subtle increases across both IgG2 against the conserved ST (*P* ≤ 0.05), as well as IgG3 responses against the novel S1 (*P* ≤ 0.01), respectively.

Diverse differences in salivary antibodies were also detected following the third antigen exposures between COVID-19–recovered (1 × prior infection + 2 × BNT162b2) and vaccinated only (2 × BNT162b2 + 1 × mRNA booster) cohorts (Figure 2, D and E). In contrast with their first mRNA vaccination, there was a decline in salivary IgA response among the COVID-19–recovered vaccinees after their second mRNA vaccination (Figure 1C and Supplemental Figure 4A). This supports previous observations that salivary IgA responses dip after the second IM vaccine dose and highlights a potential need for repeated mucosal antigen exposures to induce or maintain robust local IgA responses (6). Conversely, salivary total IgG levels remained higher in the COVID-19–recovered cohort (1 × prior infection + 2 × BNT162b2) than the vaccinated only cohort (2 × BNT162b2 + 1 × mRNA booster) after 3 antigen exposures, largely driven by IgG1 responses (Figure 1C and Supplemental Figure 4B). More importantly, COVID-19–recovered vaccinees still induced better antibody-mediated Fc engagement than vaccinated only vaccinees across multiple spike antigens (*P* ≤ 0.05) (Figure 1C; Figure 2, E and F; and Supplemental Figure 4B).

In contrast, salivary IgG subclass responses in vaccinated only vaccinees (2 × BNT162b2 + 1 × mRNA booster) (Figure 1C; Figure 2, E and F; and Supplemental Figure 4B) once again mimicked that observed in plasma after 3 antigen exposures, with strongly elevated IgG2 (*P* ≤ 0.05) and IgG4 (*P* ≤ 0.001) responses detected across multiple SARS-CoV-2 spike antigens, particularly the more conserved S2 and ST (Supplemental Figure 5, A–C). Rises in IgG2 and particularly IgG4 responses in blood after mRNA vaccination have been previously described following repeated mRNA vaccinations and were instead absent following repeated vaccinations with adenoviral vectors (13, 14). As such, here we also tested the salivary responses in vaccinated only vaccinees who had received 2 adenoviral vector vaccines prior to their mRNA booster (2 × ChAdOx1 nCoV-19 + 1 × mRNA booster; total 3 × antigen exposures) (Supplemental Figure 1A and Supplemental Figure 6A). Unsurprisingly, while the mRNA booster enhanced total IgG levels in saliva, it did not induce a detectable IgG4 response, mimicking that observed in plasma (Supplemental Figure 6, B and C).

IgG subclass switching to IgG2 and subsequently IgG4 is often thought to be a compensatory mechanism against over-inflammation due to their relatively poor ability to engage FcRs (15). Irrgang et al. recently demonstrated that the rise in IgG4 antibodies after repeated COVID-19 vaccinations coincided with a decrease in SARS-CoV-2–specific antibody-mediated functional responses in blood (14). Here, while we did detect negative correlations between both IgG2/IgG4 responses against Fcγ2aR/Fcγ3aR engagements in plasma from vaccinated only vaccinees after their mRNA boosters (2 × BNT162b2 + 1 × mRNA booster) (Supplemental Figure 5, D and E), this pattern was not replicated in paired saliva samples (Supplemental Figure 4, C and D). This suggests that the presence of elevated IgG2 and IgG4 in saliva might not be sufficient to dampen antibody-mediated FcγR engagements at the mucosa in vaccinees receiving multiple mRNA vaccines.

To corroborate the mucosal antibody signatures observed in saliva following vaccination, we further explored if similar antibody signature would also be detected in tear fluid. Despite being found in different mucosal sites, both tear fluid and saliva serve as the first line of defense against aerosolized SARS-CoV-2. Similar to salivary antibody responses, only 3 doses of mRNA vaccines induced elevated anti-spike IgG4 levels in tear fluid (Figure 2G and Supplemental Figure 7A). Vaccinees with prior COVID-19 infection also had higher levels of total IgG and FcγR3a responses in tear fluid after 3 antigen exposures (1 × prior infection + 2 × BNT162b2) (Figure 2G and Supplemental Figure 7A). Additionally, mucosal IgA responses in tear fluid of COVID-19–recovered vaccinees rose after the first vaccine dose (*P* ≤ 0.05) (Supplemental Figure 7B). These findings suggest that mucosal antibody responses to IM vaccination are conserved across different mucosal sites.

### Prior COVID-19 infection induces weak salivary neutralization of RBD–angiotensin-converting enzyme 2 interactions.

Neutralizing activity is key in both protecting from SARS-CoV-2 infection and preventing severe disease (16). Mucosal neutralizing antibodies have been shown to protect against viral challenge and are the goal of COVID-19 mucosal vaccines currently in development (17). Salivary neutralizing activity can be detected in individuals with hybrid immunity, although it may be more limited than responses in plasma and more technically challenging to measure (18). Here, we compared the ability of plasma and salivary antibodies from our vaccinated only and COVID-19–recovered cohorts to inhibit angiotensin-converting enzyme 2 (ACE2) binding to a series of RBDs, including ancestral SARS-CoV-2 and the various variants of concern (VoCs). This RBD-ACE2 surrogate assay correlates well with cell-based live virus microneutralization assay, while avoiding cell-based complications arising from the use of nonsterile saliva, making it suitable for interrogating our saliva samples (1, 19).

**Figure 3:**
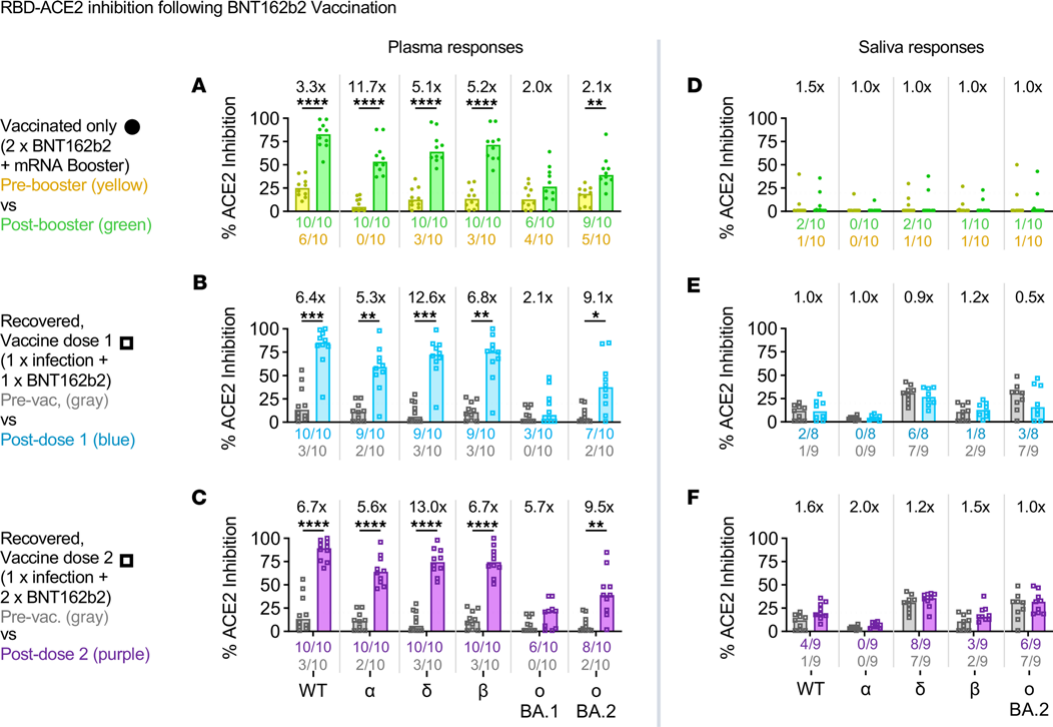
Improved salivary antibody-neutralizing activity from COVID-19–recovered vaccinees. Bar graphs depict the plasma (**A**–**C**) and salivary (**D**–**F**) inhibition of RBD-ACE2 interactions against the ancestral wild-type (WT) SARS-CoV-2 or the VoCs (α, Alpha; δ, Delta; β, Beta; σ BA.1, Omicron BA.1; σ BA.2, Omicron BA.2) by vaccinated only (*n* = 10) (**A** and **D**) and COVID-19–recovered individuals (*n* = 10) (**B**, **C**, **E**, and **F**). Fold-changes listed above the bar graphs were calculated for post-booster (green) and postvaccination responses (blue, purple) over their respective pre-booster (yellow) and prevaccination responses (gray) for each cohort and antigen. The numbers of individuals with detectable responses above the assay threshold (arbitrary 20%; dotted line) at either time point were listed under the bar graphs in their respective colors. Significant differences between both time points were calculated using the 2-tailed Mann-Whitney *U* test, followed by Bonferroni-Dunn’s test for multiple comparisons. Where significant or trending significance, *P* values were reported (*P ≤ 0.05; **P ≤ 0.01; ***P ≤ 0.001; ****P ≤ 0.0001).

Following an mRNA booster, plasma from vaccinated only vaccinees (2 × BNT162b2 + 1 × mRNA booster) strongly inhibited ACE2 binding to both ancestral and pre-Omicron VoC RBDs (*P* ≤ 0.0001) (Figure 3A). Inhibition of ACE2 binding to Omicron BA.1 and BA.2 RBDs was much more modest (Omicron BA.1: not significant; Omicron BA.2: *P* ≤ 0.01). Similarly, plasma from COVID-19–recovered vaccinees after their first mRNA vaccine showed robust inhibition of ACE2 binding to RBDs from both ancestral and pre-Omicron VoCs (*P* ≤ 0.01) (Figure 3B). However, consistent with the vaccinated only cohort, Omicron BA.1 and BA.2 RBD-ACE2 inhibition responses in plasma were weaker (Omicron BA.1: not significant; Omicron BA.2: *P* ≤ 0.05), with minimal improvement noted even after the second mRNA vaccine (Omicron BA.1: not significant; Omicron BA.2: *P* ≤ 0.01) (Figure 3C).

Expectedly, we did not find meaningful RBD-ACE2 inhibitory activity in saliva from vaccinated only vaccinees even after their mRNA booster (2 × BNT162b2 + 1 × mRNA booster; 1%–2% median inhibition across WT and VoCs) (Figure 3D), despite detectable total IgG and IgA antibodies against RBD (Figure 1C and Supplemental Figure 4B) (1). In contrast, although we detected weak RBD-ACE2 inhibitory activity in the saliva from COVID-19–recovered vaccinees even prior to vaccination, these responses did not improve significantly, even after the second mRNA vaccine dose (1 × prior infection + 2 × BNT162b2) (Figure 3, E and F). These findings support the notion that salivary neutralizing antibodies are induced following local antigen exposure at the mucosa but not by IM mRNA vaccination alone.

### Broad cross-reactivity in mucosal IgA against VoC STs.

**Figure 4:**
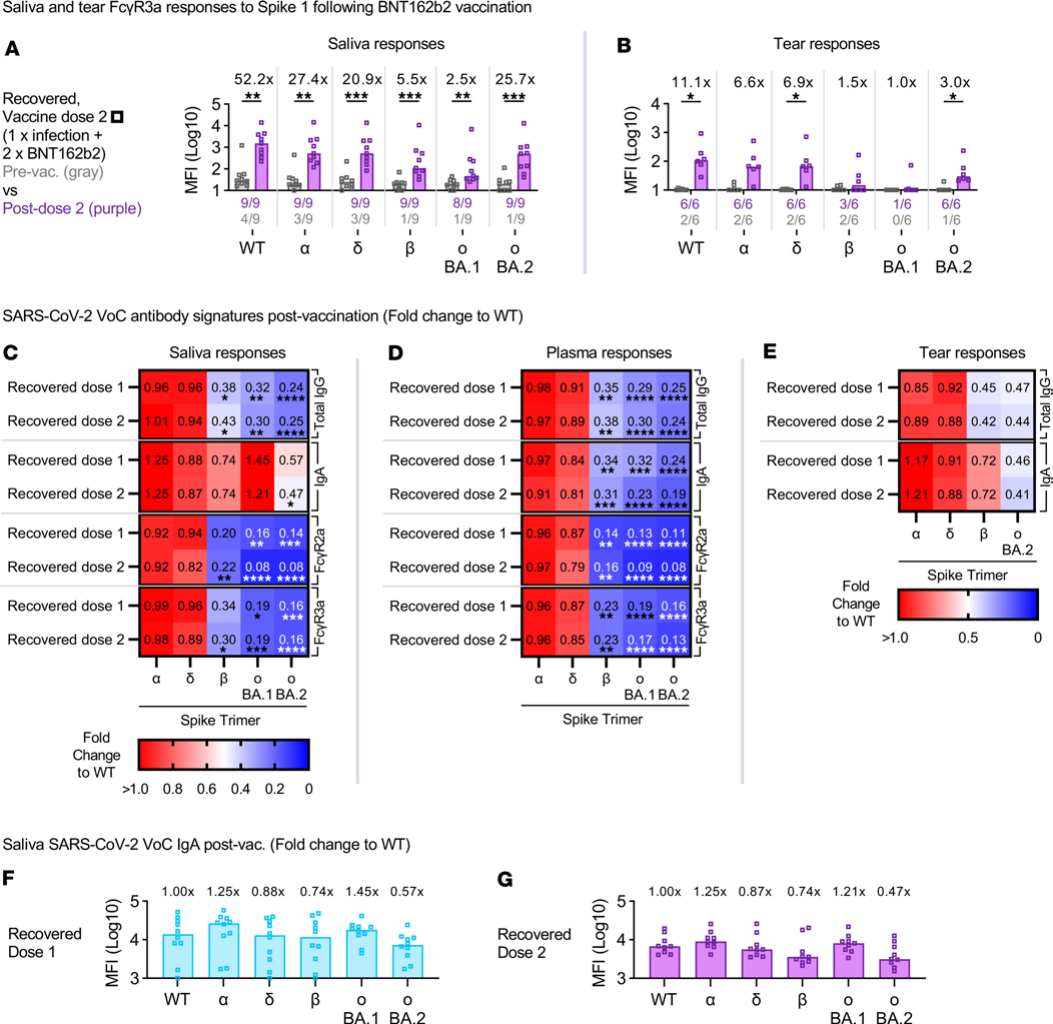
Ancestral imprinting limits cross-reactive responses against VoCs elicited by COVID-19 mRNA vaccination. Bar graphs display the salivary (*n* = 9) (**A**) and tear (*n* = 6) (**B**) Fcγ3a responses against WT SARS-CoV-2 or the VoC spike 1 antigens in COVID-19–recovered individuals following 2 doses of mRNA vaccines. Fold-changes listed above the bar graphs were calculated for postvaccination responses (purple) over their respective prevaccination responses (gray) for each antigen. The numbers of individuals with detectable responses above the assay threshold (dotted line; pre-pandemic or uninfected, unvaccinated healthy control average) at either time point were listed under the bar graphs in their respective colors. Significant differences between both time points were calculated using the 2-tailed Mann-Whitney *U* test, followed by Bonferroni-Dunn’s test for multiple comparisons. Heatmaps illustrate the VoC-specific Spike trimer (ST) salivary (*n* = 10) (**C**), plasma (*n* = 10) (**D**), and tear (*n* = 6) (**E**) antibody responses postvaccination (dose 1 or 2) for COVID-19–recovered individuals. Bar graphs describe VoC-specific ST salivary IgA responses in COVID-19–recovered individuals (*n* = 10) after 1 (**F**) or 2 (**G**) vaccine doses. The median antibody response for each VoC spike was described as a fold-change to the WT spike. Statistical significance was calculated using Friedman’s test followed by Dunn’s test for multiple comparisons and where significant or trending significance, *P* values were reported (*P ≤ 0.05; **P ≤ 0.01; ***P ≤ 0.001; ****P ≤ 0.0001).

Despite the low levels of mucosal neutralizing antibodies, we have shown above that antibodies mediating FcγR engagement were detectable in saliva and tear fluid, particularly in COVID-19–recovered vaccinees after their second mRNA vaccine (1 × prior infection + 2 × BNT162b2) (Figure 1C). These FcγR responses in saliva and tear fluid were enhanced in COVID-19–recovered vaccinees after their second mRNA vaccination and could target a range of VoC spike 1 antigens (Figure 4, A and B). However, it was also noticeable that despite similar levels of prevaccination responses, the largest gains after ancestral vaccination were unsurprisingly ancestral centric mucosal humoral responses (Figure 4, A and B).

SARS-CoV-2 antibody breadth has been shown to passively increase 1 year following SARS-CoV-2 infection as a result of continued evolution of anti–SARS-CoV-2 antibodies targeting the viral spike (20). However, repeatedly exposing COVID-19–recovered vaccinees to the ancestral antigen through vaccination could bias ancestral centric responses and diminish efforts toward developing broader antibody responses capable of recognizing newer VoCs. To explore this, we compared the systemic and mucosal humoral responses made by COVID-19–recovered vaccinees after their first and second mRNA vaccine doses. Antibody responses to the respective VoC STs were quantified, and their relative abundance was compared against the ancestral WT spike (fold-change over WT).

Broadly, the abundance in cross-reactive antibody responses against the various VoCs followed their hierarchy of escape mutations. Cross-reactive total IgG and FcγR responses against the much conserved Alpha and Delta STs were mostly comparable with the ancestral WT ST in both plasma and saliva (red on heatmap) (Figure 4, C and D). However, even more strikingly, the relative abundance of cross-reactive total IgG against the less conserved Omicron (BA.1, BA.2) (blue on heatmap) remained smaller than the more conserved Alpha and Delta VoCs (red on heatmap) in both plasma (Omicron BA.1: 0.29, 0.30; Omicron BA.2: 0.25, 0.24; doses 1 and 2; *P* ≤ 0.0001) and saliva (Omicron BA.1: 0.32, 0.30; Omicron BA.2: 0.24, 0.25; doses 1 and 2; *P* ≤ 0.01) after vaccination (Figure 4, C and D). The relative spread of cross-reactive plasma and salivary FcγR responses, as well as plasma IgA against Omicron, also largely followed a similar trend across all cohorts following vaccination (Figure 4, C and D). As such, while vaccination with the ancestral WT spike did increase SARS-CoV-2–specific antibodies, these were largely biased toward ancestral centric responses.

To the contrary, salivary IgA from COVID-19–recovered vaccinees displayed a much broader range of cross-reactivity against Beta and Omicron (BA.1, BA.2) STs after the first and second mRNA vaccines (Figure 4, C, F, and G). This trend of larger abundance in cross-reactive mucosal IgA capable of targeting Omicron was also corroborated in our analysis with tear fluid from COVID-19–recovered vaccinees following their first and second mRNA vaccines (Figure 4E). As such, the stimulation of broadly cross-reactive mucosal IgA could be key in establishing a robust protective barrier against SARS-CoV-2 infections at the mucosa by the newly emerging VoCs.

### Salivary IgA and FcγR3a antibody-mediated responses are enhanced during breakthrough infections.

**Figure 5:**
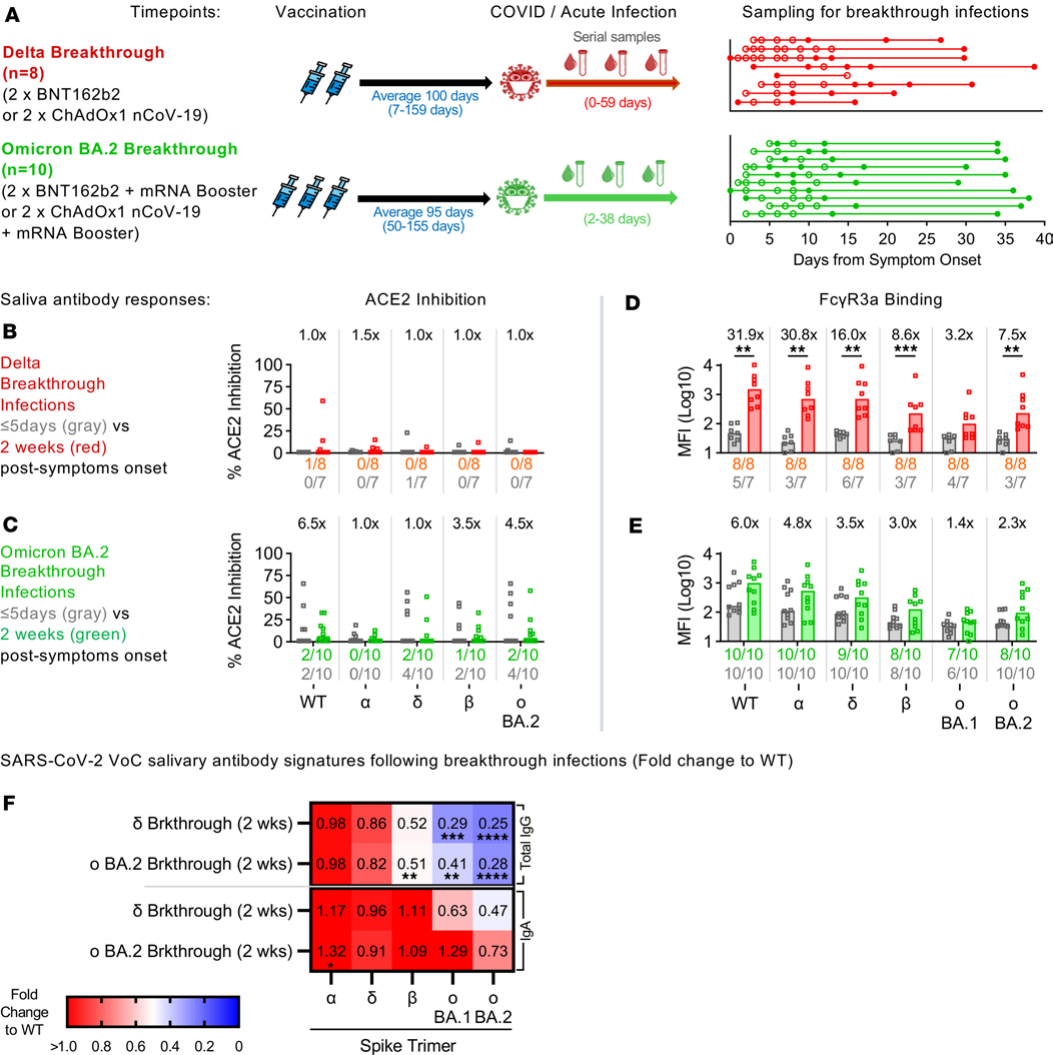
COVID-19 breakthrough infections induce salivary FcγR engagement and IgA responses. Paired saliva and plasma samples were serially collected from individuals over the course of their Delta (*n* = 8) or Omicron BA.2 breakthrough infections (*n* = 10) (**A**). Circles depict the time points where serial paired plasma and saliva samples were collected in the presence (open circles) or absence (closed circles) of a nasal swab sample. Bar graphs describe the salivary (**B** and **C**) inhibition of RBD-ACE2 interactions against the ancestral WT SARS-CoV-2 or the VoCs (α, Alpha; δ, Delta; β, Beta; σ BA.1, Omicron BA.1; σ BA.2, Omicron BA.2) by individuals with Delta (*n* = 8) (**B**) and Omicron BA.2 breakthrough (*n* = 10) (**C**) infections. Similarly, bar graphs depict the engagement of FcγR3a by salivary (**D** and **E**) antibodies by individuals with Delta (*n* = 8) (**D**) and Omicron BA.2 breakthrough (*n* = 10) (**E**) infections. Fold-changes listed above the bar graphs were calculated for responses 2 weeks after symptom onset (Delta: red; Omicron BA.2: green) over respective responses earlier during infection (gray; ≤5 days after symptom onset) for each cohort and antigen. The numbers of individuals with detectable responses above the assay threshold (dotted line) (RBD-ACE2: arbitrary 20%; FcγR3a: uninfected, unvaccinated healthy control average) at either time point were listed under the bar graphs in their respective colors. Significant differences between both time points were calculated using the 2-tailed Mann-Whitney *U* test followed by Bonferroni-Dunn’s test for multiple comparisons. Heatmap illustrates the VoC-specific ST salivary antibody responses for Delta (*n* = 8) and Omicron BA.2 breakthrough (*n* = 10) cohorts 2 weeks after symptom onset (**F**). The median antibody response for each VoC spike was described as a fold-change from the WT spike. Statistical significance was calculated using Friedman’s test followed by Dunn’s test for multiple comparisons. Where significant, *P* values were reported (*P ≤ 0.05; **P ≤ 0.01; ***P ≤ 0.001; ****P ≤ 0.0001).

Given that prior COVID-19 infection could influence both systemic and, more importantly, mucosal humoral responses following vaccination, we next wanted to explore how prior vaccination impacted systemic and mucosal humoral responses during COVID-19 breakthrough infections. To address this, we expanded our studies to breakthrough COVID-19 cohorts and collected serial saliva and plasma samples from vaccinated individuals with acute COVID-19 from 2 VoC waves (Delta, Omicron BA.2) in Victoria, Australia (Figure 5A, cohort information described in Supplemental Figure 8A and Supplemental Table 2) (8, 21, 22).

SARS-CoV-2 viral load (nasal swabs) from both VoC waves had comparable viral loads as previously reported (Supplemental Figure 8, B and C), which titered out by 2 weeks (8). As such, we compared antibody responses up to 2 weeks, to determine factors that could be associated with viral clearance. Surprisingly, despite having high detectable viral loads, only a minority of individuals with breakthrough infections developed neutralizing antibodies in their saliva (Figure 5, B and C, and Supplemental Figure 8, D and E). Most individuals with breakthrough infections failed to produce a response above our 20% assay cutoff, regardless of breakthrough wave (Delta, Omicron BA.2) (Figure 5, B and C, and Supplemental Figure 8E).

To study FcγR engagement, we focused on FcγR3a responses as we had previously noted that they were stronger and less impacted by ancestral imprinting than FcγR2a (Figure 4C). In contrast to neutralizing antibodies, most individuals with Delta or Omicron BA.2 COVID-19 breakthrough infections had detectable FcγR engagement responses in saliva (Figure 5, D and E, and Supplemental Figure 8F). Individuals with Delta breakthrough infections had 2 doses of COVID-19 vaccines and displayed low levels of cross-reactive salivary antibodies against the various VoC spikes during early infection, with only about half of the cohort recording responses above uninfected, unvaccinated healthy controls (dotted line) (Figure 5D and Supplemental Figure 8F). However, after 2 weeks, salivary antibody-mediated FcγR engagements were robustly enhanced against the Delta variant spike (16-fold increase, *P* ≤ 0.01) (Figure 5D and Supplemental Figure 8F). The effects of ancestral imprinting were also noticeable, with the ancestral WT (31.9-fold increase, *P* ≤ 0.01) and more conserved Alpha variant spikes (30.8-fold increase, *P* ≤ 0.01) gaining the largest increases in FcγR responses 2 weeks after symptom onset (Figure 5D and Supplemental Figure 8F).

In contrast, all individuals with Omicron BA.2 breakthrough infections had their mRNA boosters (3 × vaccine doses) and displayed higher levels of preexisting salivary antibodies capable of engaging FcγR early in infection as compared with the Delta breakthrough cohort (Figure 5, D and E, and Supplemental Figure 8F). Smaller fold-increases were observed for Omicron BA.2 infections 2 weeks after symptom onset (Omicron BA.2: 2.3-fold increase), with the largest differences still being ancestral centric responses (WT: 6-fold increase) (Figure 5E and Supplemental Figure 8F). The Omicron BA.2 breakthrough cohort also achieved an overall lower maximal FcγR response across all variants tested as compared with that observed with Delta breakthrough infections (Figure 5, D and E, and Supplemental Figure 8F).

Furthermore, while the relative abundance of salivary total IgG responses remained largely ancestral centric, there was a wider spread of cross-reactive salivary IgA responses that were elicited in both breakthrough cohorts after 2 weeks of symptoms (Figure 5F). This supports our observation that while levels of total IgG were associated with viral clearance in the systemic compartment (plasma) (Supplemental Figure 9, A and B), IgA levels correlated better with viral clearance in the mucosal compartment (saliva), particularly with Omicron BA.2 breakthrough infections (Supplemental Figure 9, C and D).

Interestingly, we also noted that most individuals with Delta breakthroughs who had previously received 2 × BNT162b2 vaccines displayed more robust IgG4 responses in both saliva and plasma as compared with individuals who instead received 2 × ChAdOx nCov-19 (Supplemental Figure 10, A–D). While a similar trend was observed for the Omicron BA.2 breakthroughs, it should be noted that some individuals who had received 1 × mRNA booster after 2 × ChAdOx nCov-19 also induced robust IgG4 responses, particularly against the WT ancestral antigen (Supplemental Figure 10, E–H). Unfortunately, due to the limited cohort size, we are unable to ascertain if these changes in IgG4 responses ultimately impacted FcγR engagement.

Taken together, our mucosal data suggest that despite limited neutralizing activity against novel SARS-CoV-2 antigens, salivary antibodies targeting FcγR engagement, as well as salivary IgA, could play key roles for localized cross-reactive protection at the mucosa. However, ancestral centric preexisting immunity may also influence the type and magnitude of salivary FcγR engagement antibody responses made during breakthrough infections.

### Preexisting vaccine immunity modulates systemic neutralizing and FcR antibody responses during breakthrough infections.

**Figure 6:**
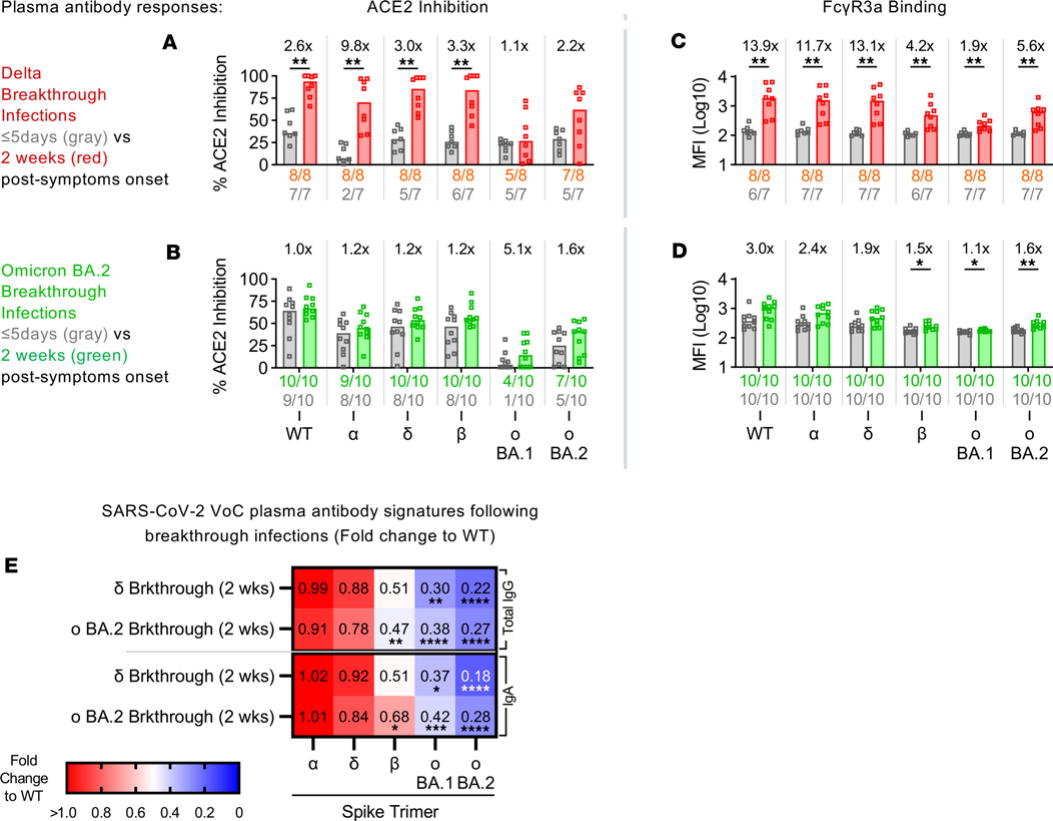
Preexisting vaccine-induced immunity modulates variant-specific plasma antibody responses during breakthrough infections. Bar graphs depict the plasma (**A** and **B**) inhibition of RBD-ACE2 interactions against the ancestral WT SARS-CoV-2 or the VoCs (α, Alpha; δ, Delta; β, Beta; σ BA.1, Omicron BA.1; σ BA.2, Omicron BA.2) by individuals with Delta (*n* = 8) (**A**) and Omicron BA.2 breakthrough (*n* = 10) (**B**) infections. Similarly, bar graphs describe the engagement of FcγR3a by plasma (**C** and **D**) antibodies by individuals with Delta (*n* = 8) (**C**) and Omicron BA.2 breakthrough (*n* = 10) (**D**) infections. Fold-changes listed above the bar graphs were calculated for responses 2 weeks after symptom onset (Delta: red; Omicron BA.2: green) over respective responses earlier during infection (gray; ≤5 days after symptom onset) for each cohort and antigen. The numbers of individuals with detectable responses above the assay threshold (dotted line) (RBD-ACE2: arbitrary 20%; FcγR3a: uninfected, unvaccinated healthy control average) at either time point were listed under the bar graphs in their respective colors. Significant differences between both time points were calculated using the 2-tailed Mann-Whitney *U* test followed by Bonferroni-Dunn’s test for multiple comparisons. Heatmap also describes the plasma antibody responses against VoC-specific STs for Delta (*n* = 8) and Omicron BA.2 breakthrough (*n* = 10) cohorts 2 weeks after symptom onset (**E**). The median antibody response for each VoC spike was described as a fold-change from the WT spike. Statistical significance was calculated using Friedman’s test followed by Dunn’s test for multiple comparisons. Where significant, *P* values were reported (*P ≤ 0.05; **P ≤ 0.01; ***P ≤ 0.001; ****P ≤ 0.0001).

In comparison, systemic antibody responses (plasma) displayed better neutralizing activity. Individuals with Delta breakthroughs (2 × COVID-19 vaccines) started out with lower levels of cross-reactive plasma antibodies against the Delta variant RBD (29% median RBD-ACE2 inhibition) early in infection (Figure 6A and Supplemental Figure 11A). Two weeks after symptom onset, the robust generation of Delta-specific antibodies led to a 3-fold increase in RBD-ACE2 inhibition (Figure 6A and Supplemental Figure 11A). Notably, significant increases in ACE2 inhibition were also observed for the ancestral strain as well as more conserved pre-Omicron variants (Alpha, Delta, Beta) (*P* ≤ 0.01). In contrast, little difference to ACE2 inhibition was seen with the more immune-escaped Omicron BA.1 RBD (1.1-fold increase in RBD-ACE2 inhibition).

Individuals with Omicron BA.2 breakthroughs all had their mRNA boosters (3 × vaccine doses) but were still infected despite having higher levels of preexisting ancestral centric SARS-CoV-2 antibodies (65% median RBD-ACE2 inhibition against WT), highlighting the immune evasiveness of the variant (Figure 6B and Supplemental Figure 11A). Smaller increases in RBD-ACE2 inhibition (1- to 1.2-fold increases) particularly against the ancestral WT and the more conserved pre-Omicron variants (Alpha, Delta, Beta) were observed after 2 weeks, despite having a lower maximal response than that from the Delta wave (67% vs. 94% median RBD-ACE2 inhibition against WT) (Figure 6B and Supplemental Figure 11A). In contrast, the largest growth in ACE2 inhibition after 2 weeks were against the Omicron variants (Omicron BA.1: 5.1-fold increase; Omicron BA.2: 1.6-fold increase), though they were still lower than cross-reactive responses induced from the Delta breakthroughs (15% vs. 27% and 41% vs. 62% median RBD-ACE2 inhibition against Omicron BA.1 and Omicron BA.2, respectively) (Figure 6B and Supplemental Figure 11A).

Systemic FcγR engagement responses trended similar to that described above for the mucosa. During early Delta breakthrough infection, most individuals had minimal levels of antibodies capable of FcγR3a engagement, as in pre-pandemic controls (dotted line) (Figure 6C and Supplemental Figure 11B). However, after 2 weeks, significant increases to FcγR engagements were noticed across all VoCs, including Omicron (*P* ≤ 0.01). The largest increases observed were ancestral centric responses with the ancestral WT (13.9-fold increase), as well as the more conserved Alpha (11.7-fold increase) and Delta spikes (13.1-fold increase).

On the other hand, preexisting levels of FcγR engagement from samples collected early in Omicron BA.2 infections remained above those found in uninfected prevaccination controls (Figure 6D and Supplemental Figure 11B). Smaller ancestral centric fold-increases were observed for BA.2 infections 2 weeks after symptom onset, achieving an overall lower maximal response across all variants tested as compared with that observed with Delta breakthrough infections (Figure 6D and Supplemental Figure 11B). Notably, the growth in plasma FcγR responses against the range of VoCs by 2 weeks after symptom onset was also mostly smaller than those changes elicited at the mucosa (Figure 5, D and E, and Figure 6, C and D). Furthermore, IgA responses in the plasma were less cross-reactive than those observed in the mucosa (Figure 5F and Figure 6F).

Taken together, our data support the notion that higher levels of preexisting plasma antibodies could negatively influence the magnitude of systemic humoral responses elicited during breakthrough infections despite comparable viral loads as measured through nasal swabs. The effects of imprinting from the ancestral strain also appear to be more obvious with FcγR engagement responses, possibly due to the larger involvement of conserved cross-reactive antibodies, as compared with neutralization.

### Rapid recall of salivary antibodies in Omicron breakthrough compared with plasma.

Since preexisting SARS-CoV-2 immunity could modulate the magnitude of antibody responses detected 2 weeks after the onset of breakthrough infections, we next investigated if variations in antibody kinetics could explain the differential rise observed in systemic and mucosal antibodies targeting the respective spike antigens. Here, we modeled the dynamics of antibody features (total IgG, IgA, and Fcγ3aR) targeting the ancestral WT, Delta, Omicron BA.1, and Omicron BA.2 S1 and ST using serial plasma and saliva samples collected from both respective COVID-19 breakthrough cohorts for up to 40 days.

**Figure 7:**
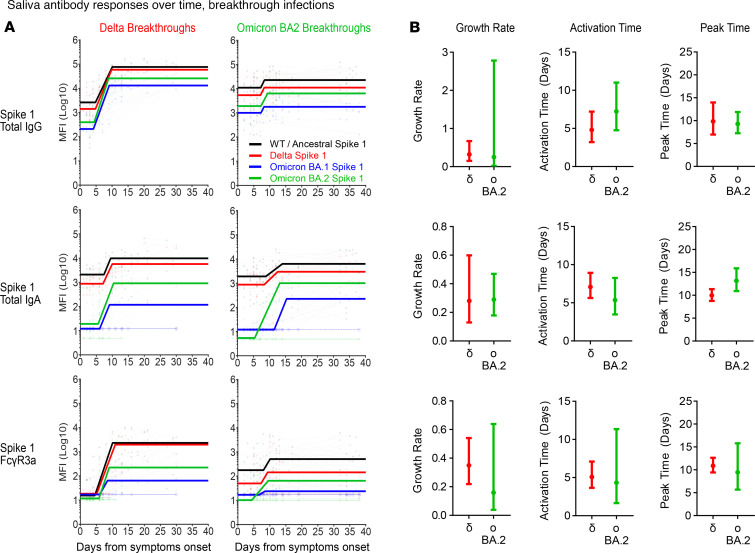
Ancestral imprinting impacts salivary antibody responses during breakthrough infections. Modeled kinetic curves (WT: black; Delta: red; Omicron BA.1: blue; Omicron BA.2: green) describe the ancestral WT and variant-specific S1 antibody responses from serially collected saliva samples during Delta (*n* = 8) or Omicron BA.2 (*n* = 10) breakthrough infections for up to 40 days after symptom onset (**A**). Connected dotted lines indicate serial samples from the same individual. Faint, thin lines with open circles at the bottom of each graph reflect samples that were excluded from the model for being below the threshold of detection (2 S.D. background readings). Dot plots displaying 95% confidence intervals beside each row of kinetic curves list the calculated growth rate, time to activation, and time to peak of variant-specific salivary responses (Delta: red; Omicron BA.2: green) by their respective breakthrough cohorts (e.g., Delta variant responses during the Delta breakthroughs) (**B**).

**Figure 8:**
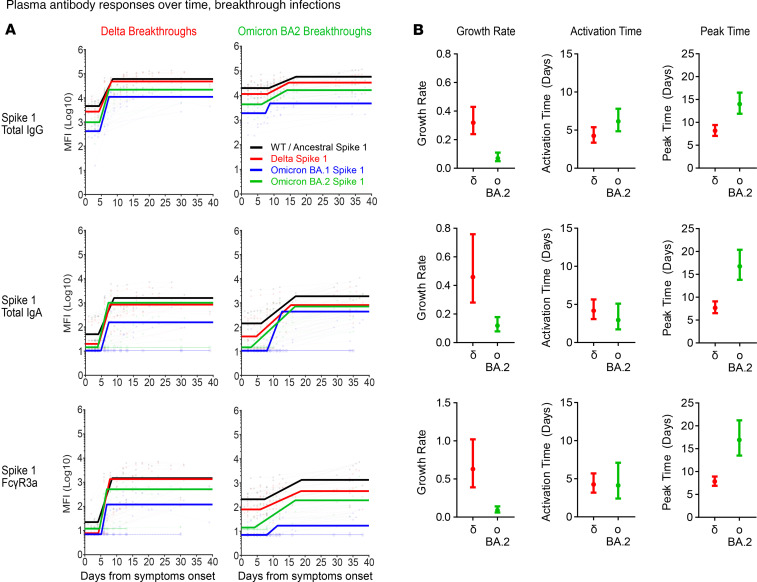
Plasma antibody responses display delayed kinetics during Omicron BA.2 breakthrough infections. Modeled kinetic curves (WT: black; Delta: red; Omicron BA.1: blue; Omicron BA.2: green) describe the ancestral WT and variant-specific S1 antibody responses from serially collected plasma samples during Delta (*n* = 8) or Omicron BA.2 (*n* = 10) breakthrough infections for up to 40 days after symptom onset (**A**). Connected dotted lines indicate serial samples from the same individual. Faint, thin lines with open circles at the bottom of each graph reflect samples that were excluded from the model for being below the threshold of detection (2 S.D. background readings). Dot plots displaying 95% confidence intervals beside each row of kinetic curves list the calculated growth rate, time to activation, and time to peak of variant-specific plasma responses (Delta: red; Omicron BA.2: green) by their respective breakthrough cohorts (e.g., Delta variant responses during the Delta breakthroughs) (**B**).

Regardless of VoC waves, antibody responses toward the ancestral WT spike (black line) largely dominated in both plasma and saliva even up to 40 days (Figure 7A, Figure 8A, Supplemental Figure 12A, and Supplemental Figure 13A). The magnitude of responses to Delta (red line), which is more similar to the ancestral WT, was also usually higher than those for Omicron (BA.1 and BA.2; blue and green lines, respectively), even for the Omicron breakthrough cohorts. These observations corroborate with our above findings that antibody responses made during acute breakthrough infection could be ancestral centric and largely driven by cross-reactive responses instead.

Despite larger variations particularly with the Omicron BA.2 responses, the growth rate, time of activation, and time of peak for salivary total IgG, IgA, and Fcγ3aR responses against the respective breakthrough variant’s S1 and ST were comparable between the Delta and Omicron BA.2 breakthrough cohorts (with overlapping 95% confidence intervals) (Figure 7B and Supplemental Figure 12B). The time of activation for plasma IgG, IgA, and Fcγ3aR responses against the respective breakthrough variant’s S1 were also comparable between the Delta and Omicron BA.2 breakthrough cohorts (with overlapping 95% confidence intervals) (Figure 8B). However, a delay in time of activation was detected in plasma IgA and Fcγ3aR responses against Omicron BA.2 ST by the Omicron BA.2 breakthrough cohort, as compared with Delta responses in the Delta breakthrough cohort (no overlapping 95% confidence intervals) (Supplemental Figure 13B).

In contrast, the growth rate for total IgG, IgA, and Fcγ3aR responses in plasma against Omicron BA.2 S1 and ST in the Omicron BA.2 breakthrough cohort (Omicron BA.2 S1: 0.07, 0.12, 0.09, respectively) was much poorer than that for Delta-specific responses in the Delta breakthrough cohort (δ S1: 0.32, 0.46, 0.63, respectively) (no overlapping 95% confidence intervals) (Figure 8B, Supplemental Figure 13B, and Supplemental Table 3). The time of peak for plasma IgG, IgA, and Fcγ3aR responses against Omicron BA.2 S1 and ST by the Omicron BA.2 breakthrough cohort (Omicron BA.2 S1: 14.01, 16.78, 16.95 days, respectively) was also much later, taking almost twice as long as that observed with Delta-specific responses in the Delta breakthrough cohort (δ S1: 8.17, 7.69, 7.85 days, respectively; no overlapping 95% confidence intervals) (Figure 8B, Supplemental Figure 13B, and Supplemental Table 3).

Finally, comparison of the growth rate and time of peak for IgG, IgA, and Fcγ3aR responses between plasma and saliva Omicron BA.2 breakthrough cohort samples identified a trend of notably slower and later Omicron BA.2 spike responses in plasma than in saliva (Figure 7B and Figure 8B). Together, the observed poorer growth rates and delays in plasma antibody responses likely account for the overall poorer magnitude of Omicron BA.2 plasma responses detected more than 2 weeks after the Omicron BA.2 breakthrough infections. These differences in antibody kinetics between both humoral compartments highlight the importance of local responses in resolving mucosal infections, particularly in the resolution of Omicron BA.2 breakthrough infections.

## Discussion

Mucosal immunity in the upper respiratory tract is a first line of defense against respiratory infections. Higher levels of salivary antibodies, especially anti-RBD secretory IgA, are associated with protection against breakthrough COVID-19 (3). Indeed, neutralizing IgA is detected in COVID-19–recovered individuals and can recognize a range of RBD mutations (23, 24). We and others have previously shown that current IM COVID-19 vaccinations are inefficient in inducing mucosal IgA responses in vaccinated only individuals (1, 5, 6). However, recent research has highlighted that COVID-19–recovered individuals (recovered, vaccinated) could induce better salivary IgA responses (2, 4).

Here, we demonstrate that COVID-19–recovered individuals generate robust salivary humoral responses after receiving their first mRNA vaccine, including an enhanced level of salivary IgA. These responses are, however, more biased toward the more conserved regions of the viral spike, namely S2, instead of the more diverse RBD. As such, not surprisingly, COVID-19–recovered individuals only induce slightly better salivary neutralizing antibodies relative to vaccinated only individuals after vaccination. However, importantly, we observed that salivary IgA from COVID-19–recovered individuals had strong and broad cross-reactivity across the range of STs from the respective SARS-CoV-2 VoCs. This highlights the importance of cross-reactive mucosal IgA in the first line of defense against new and emerging SARS-CoV-2 VoCs.

Conversely, as compared with responses after the first IM mRNA vaccination, mucosal IgA from COVID-19–recovered individuals dipped after receiving the second IM mRNA vaccine. This suggests that repeated stimulation at the mucosa, through either infection or mucosal vaccination, might be required for retaining good site-specific IgA responses (17, 25). This also aligns with evidence that receiving an additional IM booster (third dose) was not associated with much better sterilizing protection among COVID-19–recovered individuals against Omicron BA.1 infections (26).

Increasingly, studies are suggesting the role of Fc-dependent antibody effector functions in determining the outcome of SARS-CoV-2 infection, particularly in the absence of neutralizing antibodies against emerging COVID-19 variants (27). Here, we show that COVID-19–recovered individuals also induced better salivary antibody responses capable of engaging FcγRs after 2 and 3 antigen exposures, as compared with individuals with only vaccine-induced immunity. This could be due to the retention of tissue-resident memory B cells at the mucosa after COVID-19 (28).

COVID-19–recovered individuals who were subsequently vaccinated (dose 2 and 3) have been shown to develop broader cross-reactive antibody affinity maturation that can better engage Omicron subvariants than vaccinated only vaccinees (29). Here, we observed that while a second dose of mRNA COVID-19 ancestral vaccine did induce stronger overall antibody-mediated FcγR engagement in plasma and saliva, it favored responses against the imprinted ancestral WT spike. Interestingly, a recent study also showed that Omicron-specific antibodies made after receiving a single BA.5 bivalent vaccine were still largely cross-reactive to the ancestral spike (30). As such, receiving monovalent variant-based vaccines could be more effective in promoting broader cross-reactive antibodies and overcome the limitations of immune imprinting by ancestral SARS-CoV-2 spike (31, 32). Future studies should investigate if updated variant-based COVID-19 vaccines can better address newly emerging escape variants.

Irrgang et al. recently published that repeated mRNA vaccines drove the responses of noninflammatory IgG4 in circulation and that this class-switching was associated with the reduced capacity for spike-specific antibody-mediated cellular and complement responses (14). Here, we noted that salivary and tear fluid IgG responses mimicked those found in circulation, likely due to transudation, particularly in the enrichment of IgG4 responses following the third mRNA vaccination. Additional mRNA boosters may exacerbate subclass restrictions and impact the ability of salivary antibodies to better engage functional responses at the mucosa. As mRNA boosters remain a useful tool in periodically bolstering the humoral responses of vulnerable populations, future studies should evaluate if reduced mRNA vaccine dosages would be more suitable for repeated seasonal boosting of COVID-19–vaccinated populations instead.

COVID-19 vaccination and boosters have been instrumental in reducing disease susceptibility and severity in vaccinated only populations following breakthrough infections, particularly with Omicron VoCs (26). However, there have been suggestions that ancestral imprinting and antibody feedback from high levels of preexisting immunity may also restrict humoral responses during breakthrough infections (9–11, 33, 34). Havervall et al. highlighted that following Omicron BA.1 breakthrough infections, while the rise in nasal IgA responses coincided with the decline in viral load (measured by quantitative PCR, qPCR), most of the elicited IgAs were still targeting the ancestral spike instead of Omicron BA.1 (2).

Here, we demonstrated that preexisting immunity influences the magnitude of systemic neutralizing responses made following breakthrough infections. Furthermore, a single breakthrough infection appeared insufficient to boost good neutralizing responses at the mucosa in the majority of individuals studied, regardless of VoC wave (Delta, Omicron BA.1, Omicron BA.2). This gap in mucosal immunity could still leave individuals recovered from COVID-19 breakthrough infection susceptible to a repeat SARS-CoV-2 infection.

To address this immunity gap, 4 mucosal vaccines for SARS-CoV-2 (BBV154; Ad5-nCoV-IH; Sputnik V/Gam-COVID-Vac; RAZI-COV PARS) have been approved for emergency use worldwide (35–38). Our findings contrast with observations from “prime and pull” animal models, where a single intranasal booster was sufficient for the robust induction of mucosal antibodies (17, 35, 36, 38). Nevertheless, despite differences arising from infection and vaccination, our observations do align with data from human clinical trials where SARS-CoV-2–specific mucosal neutralizing antibodies were only detectable in a minority of participants receiving a single dose of mucosal vaccine (25, 37). It also remains unclear if the dampened SARS-CoV-2 breakthrough infection resulting from enhanced viral clearance by preexisting immunity may have limited viral antigen exposure required for generating better variant-specific antibodies. Future studies could address if repeated SARS-CoV-2 infections or receiving multiple variant-specific vaccines, especially mucosal vaccines, would in turn enhance mucosal humoral responses.

We also noticed a delay in the time of activation, growth rate, and time of peak of variant-specific plasma antibody responses in line with increasing preexisting immunity within the Omicron BA.2 breakthrough cohorts. It should also be noted that salivary Omicron BA.2-specific responses were quicker to peak than in plasma. This suggests that local mucosal antibody responses may play a bigger role in the timely control and clearance of mucosal infections arising from emerging COVID-19 variants.

Our data also suggest a role for IgA in supporting viral clearance during breakthrough infection. While IgA is pivotal at the mucosal barrier for keeping invading pathogens out, it has been proposed that secretory IgA could also bind and excrete pathogens across the lumen (39). This would in turn reduce viral load and modulate inflammation in the subepithelial tissues. Future studies should consider exploring this avenue of viral modulation during active infection.

Hybrid immunity has also been commonly used to refer to cohorts of either COVID-19–recovered vaccinees (recovered, then vaccinated) or individuals with breakthrough infections (vaccinated, then infected). Here, we have demonstrated that while both cohorts are conceptually similar, the order and sites of exposure, as well as their levels of preexisting immunity, can promote different humoral responses, particularly at the mucosa.

While we acknowledge that our small cohort sizes may limit the interpretations made, we hope that our serial sampling of breakthrough infections can still provide valuable insights into the early kinetics of mucosal humoral responses. Future work could be done to assess how waning levels of preexisting immunity would impact mucosal responses generated particularly against newer Omicron variants with accumulated mutations, such as XBB1.5 and XBB1.16. In addition, while we compared postvaccination responses after an equal number of antigen exposures (prior infection or vaccination), the time between antigen exposures was limited by the approved vaccination schedules in Australia (dose 2: 1 month after dose 1; booster: 5 months after dose 2). As subsequent booster shots are more evenly spaced apart, future studies would be better positioned to make fairer comparisons between the impact of antigen exposure resulting from either infection or vaccination. The impact of repeated mucosal exposures through either acquired infection or intranasal vaccination, particularly with nonancestral vaccines, on the induction of site-specific antibodies should also be explored. Furthermore, while saliva is a convenient sample for studying mucosal responses, future studies should investigate if mucosal antibody responses, particularly secretory IgA, may be further enriched in nasal fluid instead (40). We also acknowledge that collecting larger volumes of mucosal samples (saliva, tear fluid, nasal fluid) could allow the future use of cell-based live virus microneutralization assays instead.

IM COVID-19 vaccinations may be effective in generating systemic immunity to protect against severe disease, but they remain inefficient in eliciting sustained mucosal antibodies, even among COVID-19–recovered individuals. These gaps in mucosal immunity, particularly a lack of mucosal neutralizing antibodies and IgA responses, likely contribute to high rates of breakthrough infections with Omicron variants, highlighting the urgency for effective mucosal COVID-19 vaccines. While preexisting systemic immunity afforded by current COVID-19 vaccines and boosters facilitate viral clearance, more emphasis should be placed on inducing better local SARS-CoV-2–specific mucosal antibodies.

## Methods

### Cohort and sample collection.

We enrolled individuals with and without prior SARS-CoV-2 infection from a previously described cohort (41) to donate blood and saliva prior to and following vaccinations with either BNT162b2 (Comirnaty; Pfizer-BioNTech) or ChAdOx1 nCoV-19 (AstraZeneca) vaccines, as well as mRNA boosters (Supplemental Figure 1A and Supplemental Table 1).

We also recruited previously vaccinated individuals with a nasal PCR-confirmed breakthrough COVID-19 infection (8, 21, 22) during the Delta and Omicron BA.2 waves in Victoria to provide serial blood and saliva samples (Supplemental Figure 8A and Supplemental Table 2).

Whole blood was collected with sodium heparin anticoagulant, and plasma was collected and stored at –80°C until use. Saliva was collected by SalivaBio Oral Swabs (Salimetrics) and processed following manufacturer’s instructions, before being stored at –80°C until use. Basal (nonstimulated) tear samples (~7 μL per eye) were collected by capillary flow (Drummond Scientific) from the inferior tear meniscus as previously reported and also stored at –80°C until use (42).

Serially collected salivary samples had comparable levels of secretory IgA between visits (Supplemental Figure 8D). Plasma and saliva controls from COVID-19–unvaccinated, –uninfected, healthy individuals were collected on March 16, 2020, while tear controls were from pre-pandemic samples.

### Ancestral SARS-CoV-2 multiplex bead assay.

SARS-CoV-2–specific antibody isotypes (IgG, IgA, IgM) and subclasses (IgG1-4, IgA1-2) in plasma (1:1,600), saliva (1:12.5), and tear (1:25) from the respective pre-pandemic and vaccinated cohorts were assessed using a customized multiplex bead-based array consisting of 4 ancestral SARS-CoV-2 proteins, including whole ST (gift from Peter Doherty Institute for Infection and Immunity, University of Melbourne, Melbourne, Victoria, Australia), S1 (Sino Biological), S2 (Acro Biosystems), and RBD (gift from Peter Doherty Institute for Infection and Immunity, University of Melbourne, Melbourne, Victoria, Australia) as previously described (1) (Supplemental Figure 1B). SIVgp120 protein (Sino Biological) and uncoupled BSA-blocked beads were included as negative controls for background subtraction, while H1Cal2009 (Sino Biological) and tetanus toxoid (MilliporeSigma) were included as positive controls. Plasma and saliva concentrations used in the array were chosen based on a dilution series (Supplemental Figure 14, A and B). Briefly, antigen-coupled beads were incubated with the respective samples on a shaker overnight at 4°C, before being washed, then incubated with phycoerythrin-conjugated detection antibodies (Southern Biotech) (Supplemental Table 4) on a shaker for 2 hours at room temperature (RT). Beads were washed again and read on the Flexmap 3D (Luminex). Assays were repeated in duplicates.

Engagement of SARS-CoV-2–specific antibodies against FcγRs were measured using surrogate FcγR dimers (FcγR2a, CD32; FcγR3a, CD16) as previously described (gift from Burnet Institute) (43). After incubation with samples, the washed beads were first incubated with surrogate FcγR dimers on a shaker for 2 hours at RT, washed again, and then incubated with Streptavidin-R-Phycoerythrin (SAPE; Thermo Fisher Scientific) on a shaker for a further 2 hours at RT. Finally, beads were washed and read on the Flexmap 3D. Assays were repeated in duplicates.

### SARS-CoV-2 variant multiplex bead assay.

To assess plasma (1:25,600), saliva (1:12.5), and tear (1:25) antibody responses from booster and breakthrough cohorts, ancestral and variant (Alpha, Beta, Delta, Omicron BA.1, Omicron BA.2) whole ST (gift from the Peter Doherty Institute for Infection and Immunity, University of Melbourne, Melbourne, Victoria, Australia) and S1 (Sino Biological) were used to form a customized bead array (Supplemental Figure 15, A and B). SARS-CoV-2–specific total IgG and IgA responses were assessed using biotin-conjugated detection antibodies (MabTech) (Supplemental Table 4). As above, following incubation with samples, the washed beads were first incubated with the biotin-conjugated detection antibodies on a shaker for 2 hours at RT. Beads were then washed and incubated with SAPE for another 2 hours at RT, before being washed and read on the Flexmap 3D. The ability of SARS-CoV-2 variant-specific plasma (1:12,800) and saliva (1:12.5) antibodies to mediate FcγR engagements (FcγR2a, CD32; FcγR3a, CD16) was measured using the surrogate FcR dimers as described above. Assays were repeated in duplicates.

### Variant RBD-ACE2 inhibition bead assay.

Neutralizing activity of plasma (1:800, 1:4,000) and saliva (1:12.5) samples from booster and breakthrough cohorts against the SARS-CoV-2 VoCs (Alpha, Beta, Delta, Omicron) were assessed using a surrogate RBD-ACE2 inhibition assay. As previously described (1, 19), ancestral or variant RBD-coupled beads (Sino Biological) were incubated with avi-tagged biotinylated ACE2 (gift from Dale Godfrey, Nicholas Gherardin, and Samuel Redmond, the Peter Doherty Institute for Infection and Immunity, University of Melbourne) in the presence of the respective plasma and saliva samples and assay buffers (plasma: 0.1% BSA in PBS; saliva: 0.1% BSA, 1% Triton X-100 in PBS) on a shaker for 2 hours at RT. Beads were washed and then incubated with SAPE on a shaker for 1 hour at RT. R-Phycoerythrin Biotin-XX conjugate (Thermo Fisher Scientific) was then added to the beads and incubated on a shaker for a further hour at RT. Finally, beads were washed and read on the Flexmap 3D. A nominal cutoff of 20% (depicted by dotted line in figures) was also set as previously described (19). As the binding of Omicron BA.1 RBD to ACE2 greatly diminished in the presence of 1% Triton X-100, resulting in a loss of assay resolution, we opted to remove it from the RBD-ACE2 panel for saliva samples. Ancestral and variant-specific RBD total IgG and IgA responses were also assessed using biotin-conjugated detection antibodies as described above (Supplemental Figure 15A). Assays were repeated in duplicates.

### Analysis of viral RNA load by qPCR.

For viral RNA extraction, briefly, 200 μL of sample was extracted with the QIAamp 96 Virus QIAcube HT kit (QIAGEN) on the QIAcube HT System (QIAGEN) according to manufacturer’s instructions. Purified nucleic acid was then immediately converted to cDNA by reverse transcription with random hexamers using the SensiFAST cDNA Synthesis Kit (Bioline Reagents) as per manufacturer’s instructions. cDNA was used immediately in the real-time reverse transcription PCR (rRT-PCR) or stored at –20°C. A total of 3 μL of cDNA was added to a commercial real-time PCR master mix (PrecisionFast qPCR Master Mix; Primer Design) in a 20 μL reaction mix containing primers and probe (final concentration of 0.9 mM primer and 0.2 mM probe, respectively). Samples were tested for the presence of SARS-CoV-2 RNA-dependent RNA polymerase (RdRp)/helicase (Hel), spike (S), and nucleocapsid (N) genes using previously described primers and probes (44, 45). Thermal cycling and rRT-PCR analyses for all assays were performed on the ABI 7500 FAST real-time PCR system (Applied Biosystems) with the following thermal cycling profile: 95°C for 2 minutes, followed by 45 PCR cycles of 95°C for 5 seconds and 60°C for 25 seconds for N gene and 95°C for 2 minutes, followed by 45 PCR cycles of 95°C for 5 seconds and 55°C for 25 seconds for RdRp/Hel gene and S gene.

### Kinetics analysis.

We used a piecewise model to estimate the activation time and growth rate of various immune responses (total IgG, IgA, and FcγR3aV) following breakthrough infections. The response variables had background levels subtracted by taking the mean of all the background values, and the threshold for detection was set at 2 standard deviations above the background responses. The model of the immune response for individual *y* at time *i* can be written as:
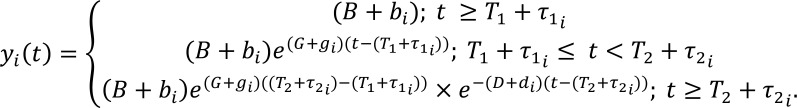


The model has 5 parameters: baseline level (*B*), growth rate (*G*), timing on onset of growth (*T_1_*), decay (*D*), and time of peak (*T_2_*). For a period before *T_1_*, we assumed a constant baseline value for the immune response (which is higher than or at the background level). After the activation time, the immune response will grow at a rate of *G* until *T_2_*. From *T_2_*, the immune response will decay at a rate of *D*. For each individual *i*, the parameters were taken from a normal distribution, with each parameter having its own mean (fixed effect). A diagonal random effect structure was used, where we assumed there was no correlation within the random effects. The model was fitted to the log-transformed data values, with a constant error model distributed around zero with a standard deviation σ. To account for the values less than the limit of detection, a censored mixed-effect regression was used to fit the model. Model fitting was performed using MonolixR2019b.

### Data normalization for multivariable multiplex analysis.

Positive control antigens (Influenza A H1N1 hemagglutinin) were removed from the database prior to analysis. Data for each detector were normalized to account for (a) background noise and (b) control antigens. First, the mean, μ_d_, of the blank wells for each detector was subtracted. After this background subtraction, values lower than 2σ_d_ (where σ_d_ is the standard deviation of the values of the blank wells for detector *d*) were assumed to be below the limit of detection. If any feature contained any negative values, the entire data set was right shifted by adding the minimum value for that feature back to all samples within that feature. Second, multiplex data were then further reduced by the (background normalized) value of the control antigen–detector pair data. Control antigens were SIVgp120 for saliva and BSA for plasma. Normalized data were log-transformed using the following equation, where x is the normalized data and y is the normalized log-transformed data: y = log10(x + 1). Data were further normalized by mean centering and variance scaling each feature using the *z* score function in MATLAB as previously described (43).

### Multivariable methods for identification of key antibody features.

A Least Absolute Shrinkage and Selection Operator (LASSO) penalized logistic regression model was used to identify the minimal set of features that differentiated between COVID-19–recovered and COVID-19 vaccinated only vaccinees (46). The feature selection stability was defined as the proportion of times that a feature was selected when the model was repeatedly fitted to 1,000 resampled subsets of data as previously described (47). PCA was then performed. Two-dimensional score plots were generated to visually assess separation between groups. All analysis was conducted using the Statistics and Machine Learning Tool on MATLAB, data were extracted, and figures were graphed using GraphPad Prism.

### Statistics.

Statistical analysis was performed with GraphPad Prism 9 (GraphPad Software). To transform the data in percentages for use in the radar plots (Figure 1C and Supplemental Figure 3A), the median of each cohort’s/time point’s antigen-specific detector-specific MFI was divided by the antigen-specific MFI from the 98th percentile for that detector (98th percentile was chosen to minimize the impact of outliers on the data transformation). Antibody levels between cohorts/time points were presented as medians and compared using 2-tailed Mann-Whitney *U* tests or Friedman’s tests, with corrections for multiple comparisons as required. Spearman’s rank correlation analyses were performed to study associations between antibody signatures. *P* ≤ 0.05 is considered significant.

### Study approval.

The study protocols were approved by the University of Melbourne Human Research Ethics Committee (2021-21198-15398-3, 2056689, 11507), and all associated procedures were carried out in accordance with approved guidelines. All participants provided written informed consent in accordance with the Declaration of Helsinki.

### Data availability.

All data are available in the supplemental tables and Supporting Data Values file.

## Author contributions

KJS and AWC were responsible for conceptualization. KJS, AWC, and SJK were responsible for data curation. KJS, AWC, SJK, PR, and ERH were responsible for methodology. KJS, AWC, SJK, PR, ERH, CWT, LFW, BDW, PMH, LED, SKD, RAP, HEK, JAJ, and AKW were responsible for investigation. KJS, AWC, SJK, AR, DC, and MPD were responsible for formal analysis. KJS was responsible for writing of the original draft and editing. AWC and SJK were responsible for review, editing, project administration, and funding acquisition. AWC was responsible for supervision.

## Supplementary Material

Supplemental data

Supporting data values

## Version 1 09/22/2023

Electronic publication
