# Supplementary material for: Preexisting immunity restricts mucosal antibody recognition of SARS-CoV-2 and Fc profiles during breakthrough infections
Source: JCI Insight. 2023 Sep 22;8(18):e172470. doi: 10.1172/jci.insight.172470 (PMC10561726; doi:10.1172/jci.insight.172470)
Supplement: Supplemental data [file jciinsight-8-172470-s115.pdf]

Supp Figures

(15 Supp Figures
+ 3 Supp Tables)

A Summary of pre-pandemic controls and vaccinated cohorts

	Pre-pandemic Controls	Comirnaty / BNT162b2 (Pfizer-BioNtech) vaccinees		ChAdOx1 nCoV-19 (AstraZeneca) vaccinees
Variables	Pre-pandemic (n=20)	Vaccinated only (n=20)	COVID-19 Recovered (Convalescent, vaccinated) (n=10)	Vaccinated only (n=17)
Age, mean (range), years	30.5 (21 – 60)	34.6 (26 – 58)	49.5 (24 – 65)	46.5 (23 – 69)
Gender				
Female (%)	16 (80.0%)	13 (65.0%)	6 (60.0%)	10 (58.8)
Male (%)	4 (20.0%)	7 (35.0%)	4 (40.0%)	7 (41.2)
Time from symptom onset till sample collection, mean (range), days			478.1 (412 – 534)	
Vaccination				
Time after 1st shot till sample collection, mean (range), days		9.1 (6 – 12)	12.5 (7 – 18)	13.4 (7 – 22)
Time between 1st and 2nd shot, mean (range), days		24.0 (21 – 33)	28.0 (21 – 48)	83 (70 – 92)
Time after 2nd shot till sample collection, mean (range), days		14.1 (12 – 22)	20.1 (10 – 34)	13.4 (7 – 22)
Time between 2nd shot and 5 months sample collection, mean (range), days		218.8 (188 – 226)		149.6 (101 – 184)
Time after 3rd shot till sample collection, mean (range), days		12.4 (10 – 15)		20.1 (10 – 31)

B Schematic of SARS-CoV-2 spike protein

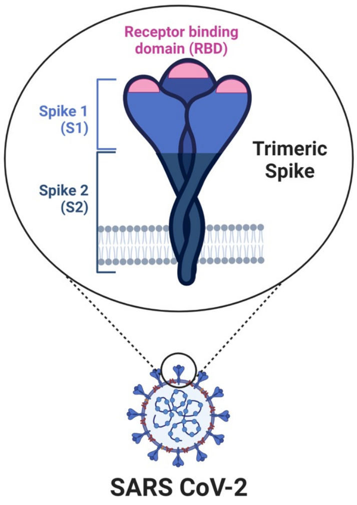

Supplementary Figure 1: Cohort Information and Spike schematic. Details of the pre-pandemic controls, vaccinated only vaccinees and COVID-19 recovered vaccinees included in the study **(A)**. Schematic of the SARS-CoV-2 Spike protein describing the four different types of Spike proteins used in the multiplex assays **(B)**.

Saliva antibody comparisons after two antigen exposures

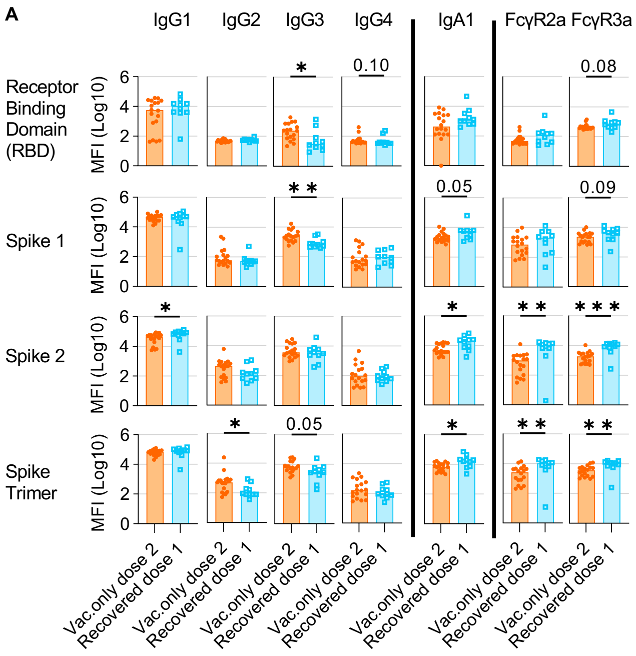

Supplementary Figure 2: Comparisons of mucosal antibody responses between vaccinated only and COVID-19 recovered vaccinees after two antigen exposures. Bar graphs describe the key differences in ancestral SARS-CoV-2 Spike-specific salivary **(A)** antibody responses between vaccinated only (*n* = 18) and COVID-19 recovered cohorts (*n* = 10) after two antigens exposures. Statistical significance was calculated using the two-tailed Mann-Whitney *U* test. Where significant or trending significance, *P*-values were reported (**P* ≤ 0.05; ***P* ≤ 0.01; ****P* ≤ 0.001; *****P* ≤ 0.0001).

Plasma antibody signatures following BNT162b2 vaccination

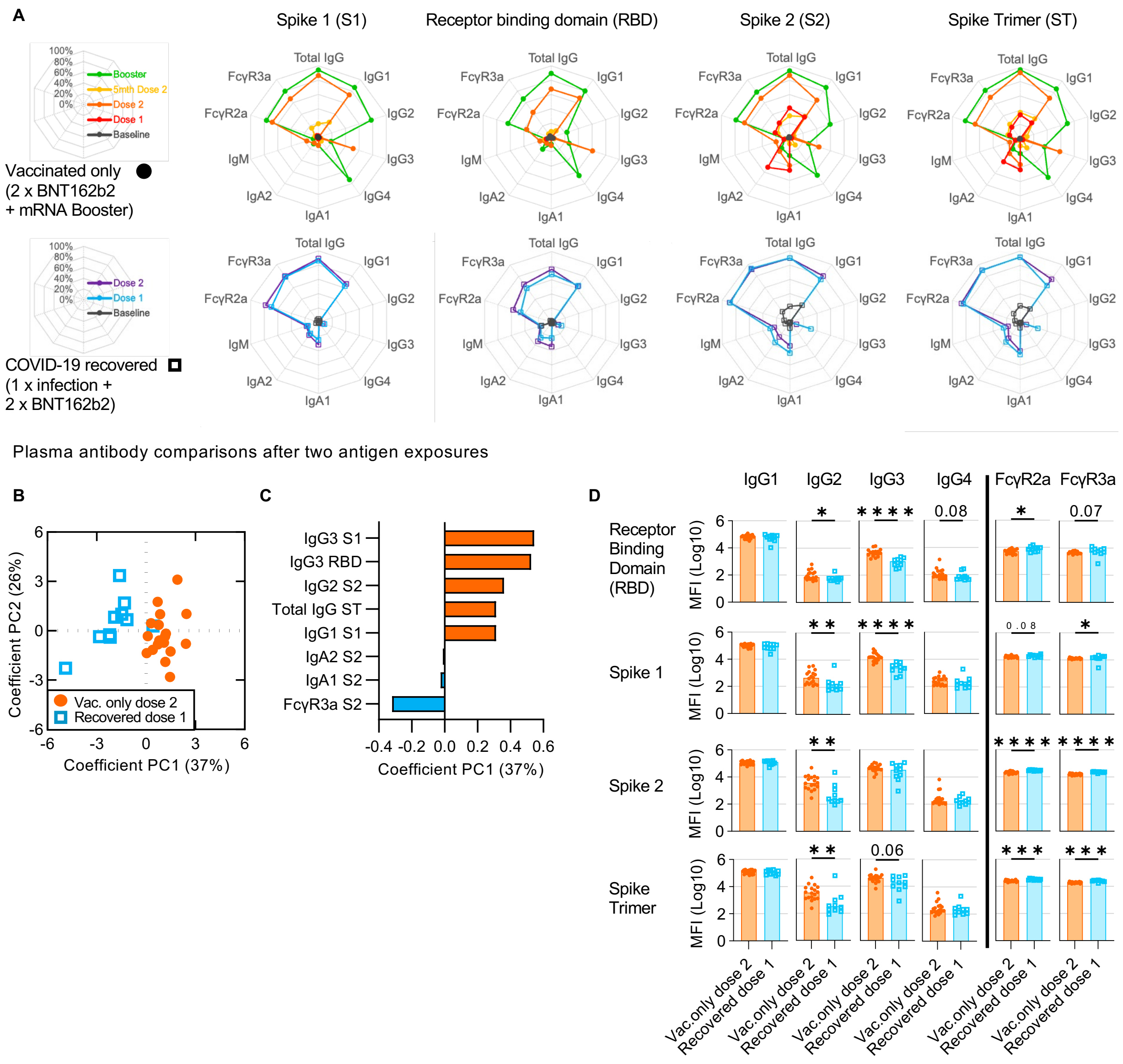

Supplementary Figure 3: Plasma antibodies from vaccinated only vaccinees show rises in IgG2 and IgG3 responses after two antigen exposures. Plasma antibody isotype and subclass responses from both cohorts against the various SARS-CoV-2 Spike antigens were compiled into respective radar plots **(A)**. The individual median antibody isotype/subclass response for each Spike antigen was transformed into percentages using the antigen-specific MFI from the 98th percentile for that detector (98th percentile was chosen to minimize the impact of outliers on the data transformation). PCA of all 40 antibody features for vaccinated only (closed circles) (n = 18) and COVID-19 recovered (open squares) (n = 10) individuals after two antigen exposures **(B)**. Loading plots and bar graphs describe the key differences between both cohorts after two **(C and D)** antigens exposure. Statistical significance was calculated using the two-tailed Mann-Whitney U test and where significant or trending significance, P-values were reported (*$P \leq 0.05$; **$P \leq 0.01$; ***$P \leq 0.001$; ****$P \leq 0.0001$).

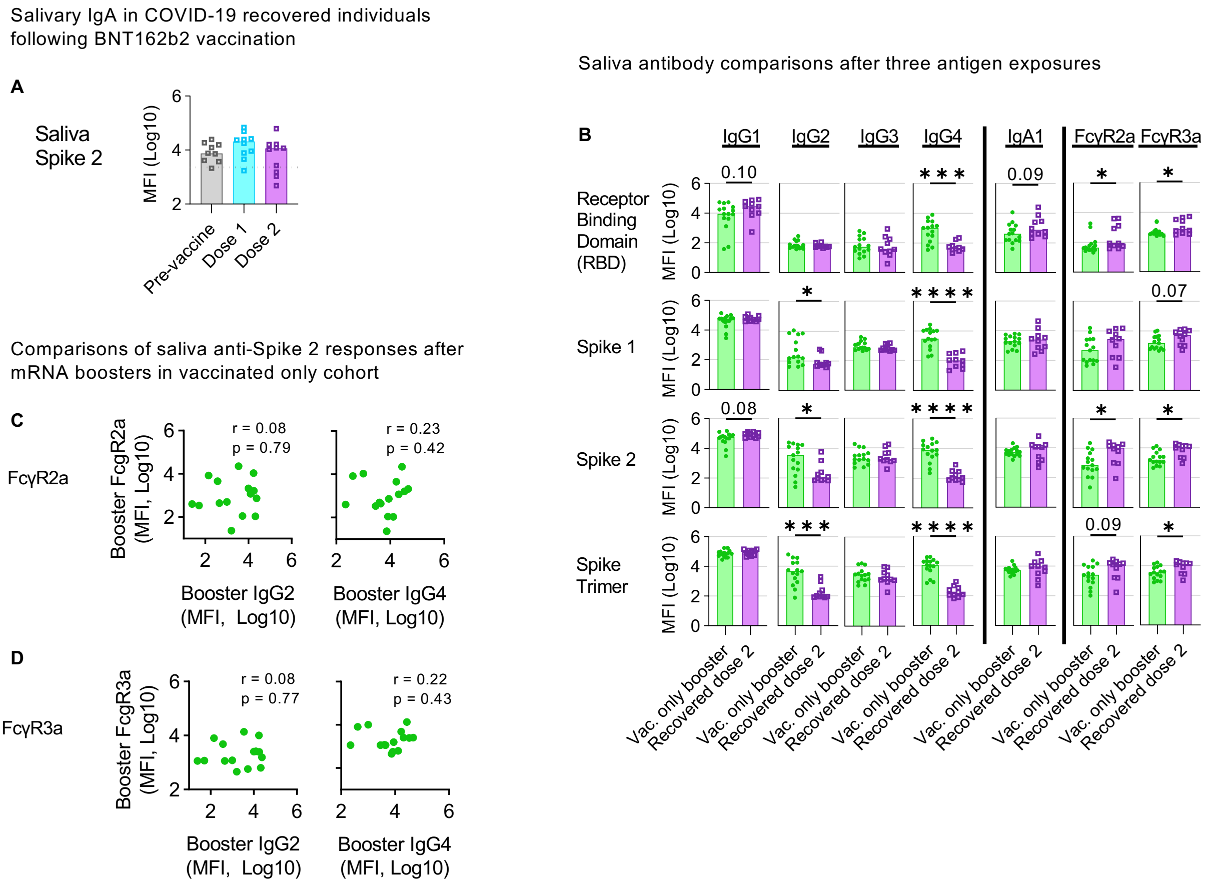

Supplementary Figure 4: Salivary antibody responses after three antigen exposures. Bar graphs illustrate the changes in anti-Spike 2 IgA responses in saliva of COVID-19 recovered individuals (*n* = 10) after their first and second mRNA vaccination **(A)**. Statistical significance was calculated using Friedman's test followed by Dunn's test for multiple comparisons. Bar graphs also describe the key differences in ancestral SARS-CoV-2 Spike-specific salivary antibody responses between vaccinated only (*n* = 15) and COVID-19 recovered cohorts (*n* = 10) after three antigens exposures **(B)**. Statistical significance was calculated using the two-tailed Mann-Whitney *U* test. Where significant or trending significance, *P*-values were reported (**P* ≤ 0.05; ***P* ≤ 0.01; ****P* ≤ 0.001; *****P* ≤ 0.0001). Spearman correlations describe salivary IgG2 and IgG4 responses against FcγR2a **(D)** and FcγR3a **(E)** engagement in the vaccinated only cohort (*n* = 20) after three mRNA vaccines.

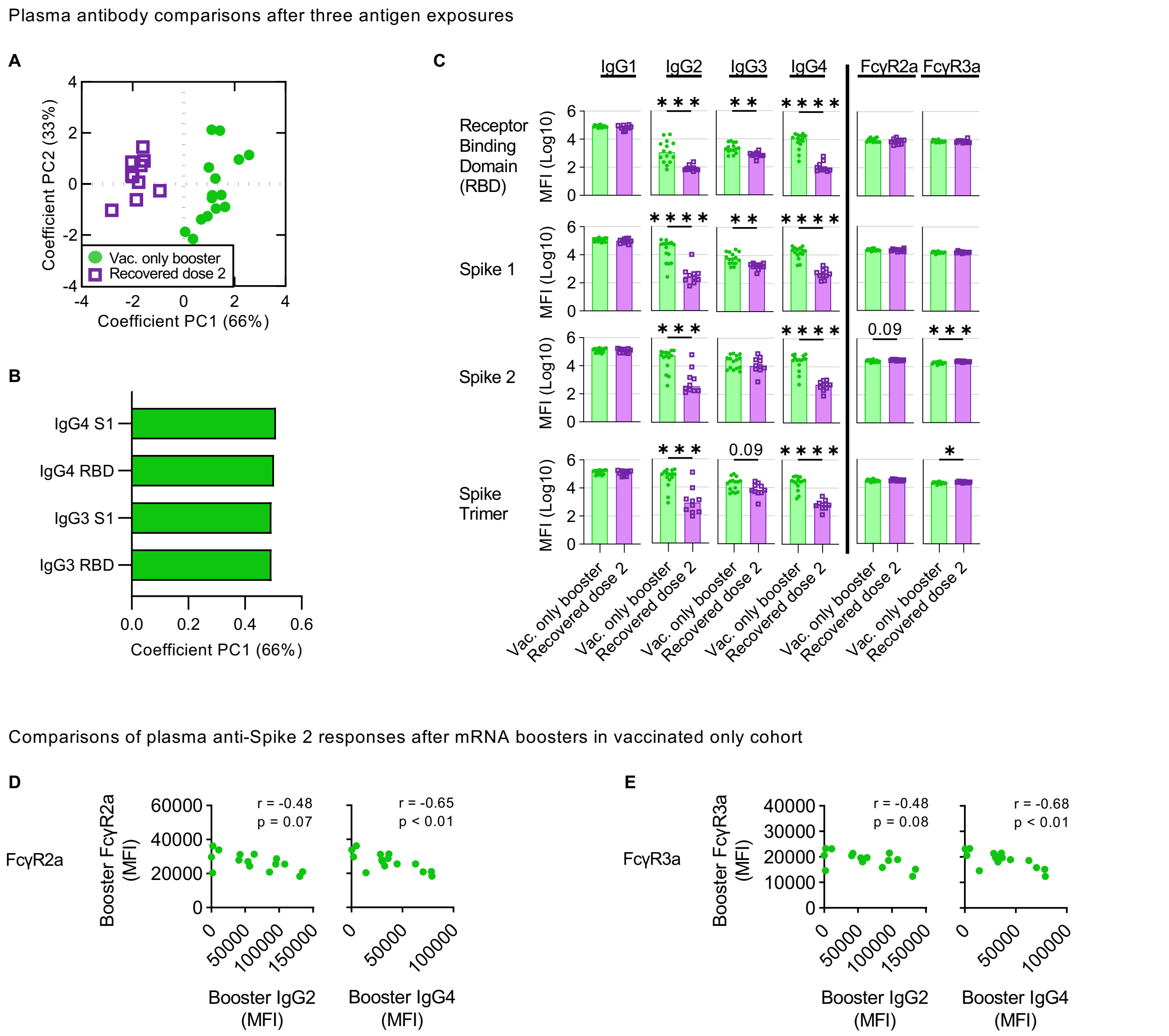

Supplementary Figure 5: Plasma antibodies from vaccinated only vaccinees show strong IgG2 and IgG4 responses after receiving third COVID-19 mRNA vaccine. PCA of all 40 antibody features for vaccinated only (closed circles) (*n* = 15) and COVID-19 recovered (open squares) (*n* = 10) individuals after three **(A)** antigen exposures. Loading plots and bar graphs describe the key differences between both cohorts after three antigen exposures **(B and C)**. Statistical significance was calculated using the two-tailed Mann-Whitney *U* test and where significant or trending significance, *P*-values were reported (**P* ≤ 0.05; ***P* ≤ 0.01; ****P* ≤ 0.001; *****P* ≤ 0.0001). Spearman correlations describe plasma IgG2 and IgG4 responses against FcγR2a **(D)** and FcγR3a **(E)** engagement in the vaccinated only cohort (*n* = 15) after three mRNA vaccines.

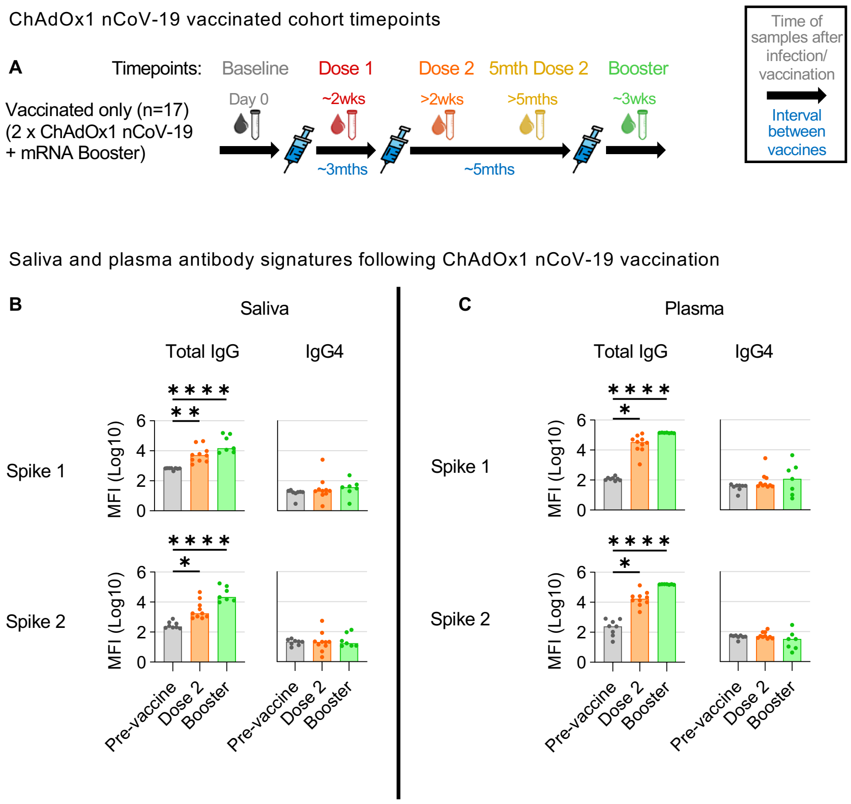

Supplementary Figure 6: No changes in salivary or plasma IgG4 responses after repeated adenoviral vector COVID-19 vaccination. Paired saliva and plasma samples were collected pre- and post-vaccination from vaccinated only vaccinees at the indicated time-points **(A)**. Bar graphs illustrate the changes in total IgG and IgG4 responses against Spike 1 and Spike 2 respectively in saliva **(B)** and plasma **(C)** of vaccinated only vaccinees (*n* = 17) after their second ChAdOx nCoV-19 vaccine (orange) or mRNA booster (green). Statistical significance was calculated using Friedman's test followed by Dunn's test for multiple comparisons. Where significant or trending significance, *P*-values were reported (**P* ≤ 0.05; ***P* ≤ 0.01; ****P* ≤ 0.001; *****P* ≤ 0.0001).

Tear antibody comparisons after three antigen exposure

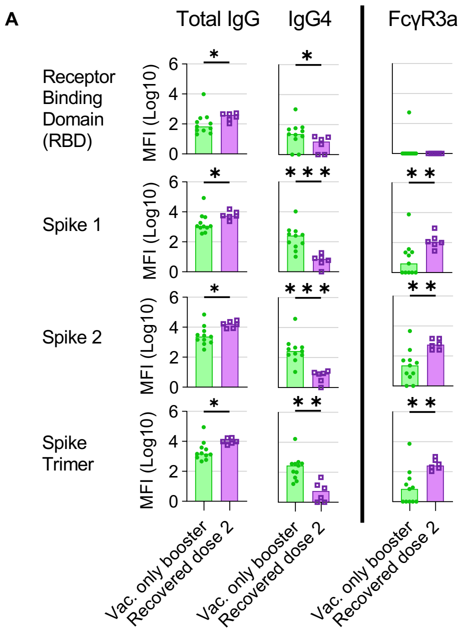

Tear IgA in COVID-19 recovered individuals following BNT162b2 vaccination

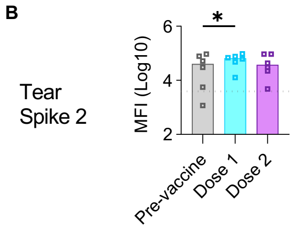

Supplementary Figure 7: Tear antibody responses following COVID-19 vaccination corroborate with salivary responses. Bar graphs describing key features in ancestral SARS-CoV-2 Spike-specific tear antibody responses between vaccinated only (*n* = 11) and COVID-19 recovered cohorts (*n* = 6) after three antigens exposures **(A)**. Statistical significance was calculated using the two-tailed Mann-Whitney *U* test. Bar graphs also illustrate the changes in anti-Spike 2 IgA responses in tear fluid **(B)** of COVID-19 recovered individuals (*n* = 6) after their first and second mRNA vaccination. Statistical significance was calculated using Friedman's test followed by Dunn's test for multiple comparisons. Where significant or trending significance, *P*-values were reported (*$P \leq 0.05$; **$P \leq 0.01$; ***$P \leq 0.001$; ****$P \leq 0.0001$).

Summary of breakthrough cohorts

A

	Delta breakthroughs	Omicron BA.2 breakthroughs
Variables	(n=8)	(n=10)
Age, mean (range), years	34.6 (19 – 58)	54.3 (40 – 66)
Gender		
Female (%)	5 (62.5%)	5 (50.0%)
Male (%)	3 (37.5%)	5 (50.0%)
Vaccination doses prior to breakthrough infection (%)	2 (100.0%)	3 (100.0%)
Time from last vaccination till breakthrough infection, mean (range), months	3.3 (0.3 – 5.3)	3.2 (1.6 – 5.3)

Breakthrough infections, viral load kinetics

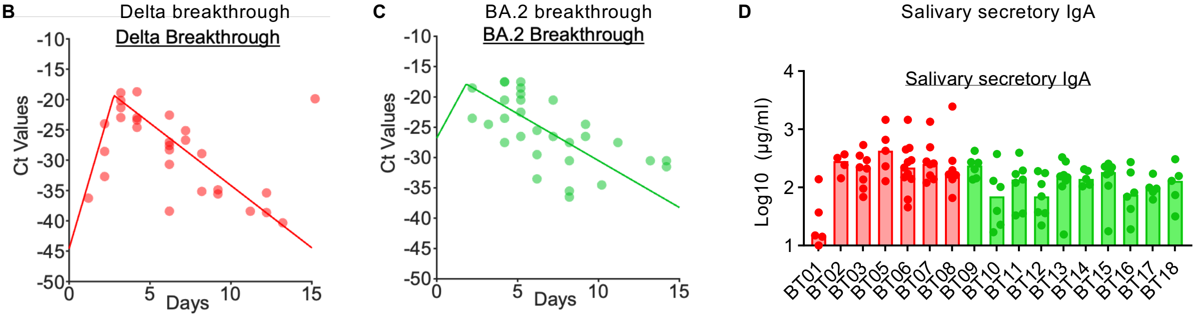

Saliva SARS-CoV-2 VoC antibody signatures following breakthrough infections

% ACE2 Inhibition (≤5 days vs 2 weeks post-onset)

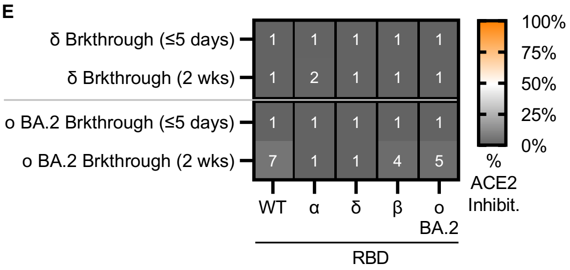

FcγR3a Responses (≤5 days vs 2 weeks post-onset)

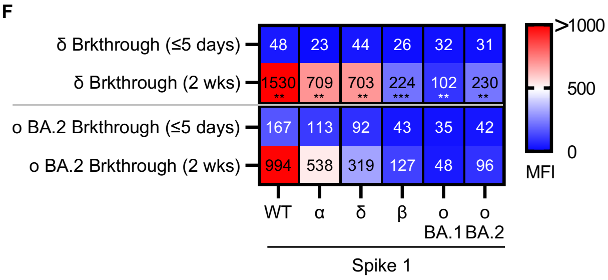

Supplementary Figure 8: Viral load kinetics of breakthrough infections and influence of pre-existing vaccine-induced immunity on elicited antibody responses. Details of vaccination status and samples collected for the COVID-19 breakthrough infection cohorts post symptom onset **(A)**. Modelled kinetic curves describe the viral load (Ct value) post-symptom onset as determined by nasal swab samples collected during the Delta (*n* = 8) **(B)** (Red) and Omicron BA.2 (*n* = 10) **(C)** (Green) COVID-19 breakthrough waves. Bar graphs show the secretory IgA present in serially collected saliva samples from COVID-19 breakthrough individuals **(D)**. Heat maps compare the salivary inhibition of RBD-ACE2 interactions against the ancestral wildtype (WT) SARS-CoV-2 or the VoCs (α, Alpha; δ, Delta; β, Beta; o BA.1, Omicron BA.1; o BA.2, Omicron BA.2) between the start and clearance of Delta (*n* = 8) and Omicron BA.2 (*n* = 10) COVID-19 breakthrough infections **(E)**. Heat maps also describe the salivary antibody responses capable of FcγR3a engagement that are elicited early and 2 weeks post-symptom onset of Delta (*n* = 8) and Omicron BA.2 (*n* = 10) COVID-19 breakthrough infections **(F)**. Significant differences between both timepoints were calculated using Friedman's test followed by Dunn's test for multiple comparisons. Where significant or trending significance, *P*-values were reported (**P* ≤ 0.05; ***P* ≤ 0.01; ****P* ≤ 0.001; *****P* ≤ 0.0001).

Breakthrough infections, comparison of antibody responses vs viral load

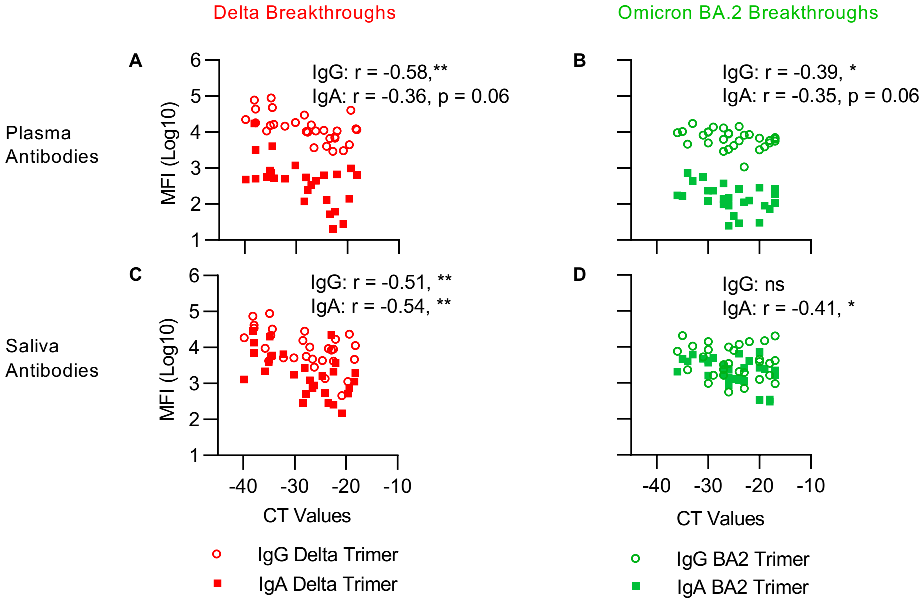

Supplementary Figure 9: Correlations of antibody responses and viral load post-breakthrough infections. Spearman correlation of plasma **(A and B)** and saliva **(C and D)** IgG (open circles) and IgA (closed squares) responses against viral load (Ct value) in the Delta (*n* = 8) **(A and C)** (Red) and Omicron BA.2 (*n* = 10) **(B and D)** (Green) COVID-19 breakthrough waves. Where significant or trending significance, *P*-values were reported (**P* ≤ 0.05; ***P* ≤ 0.01; ****P* ≤ 0.001; *****P* ≤ 0.0001).

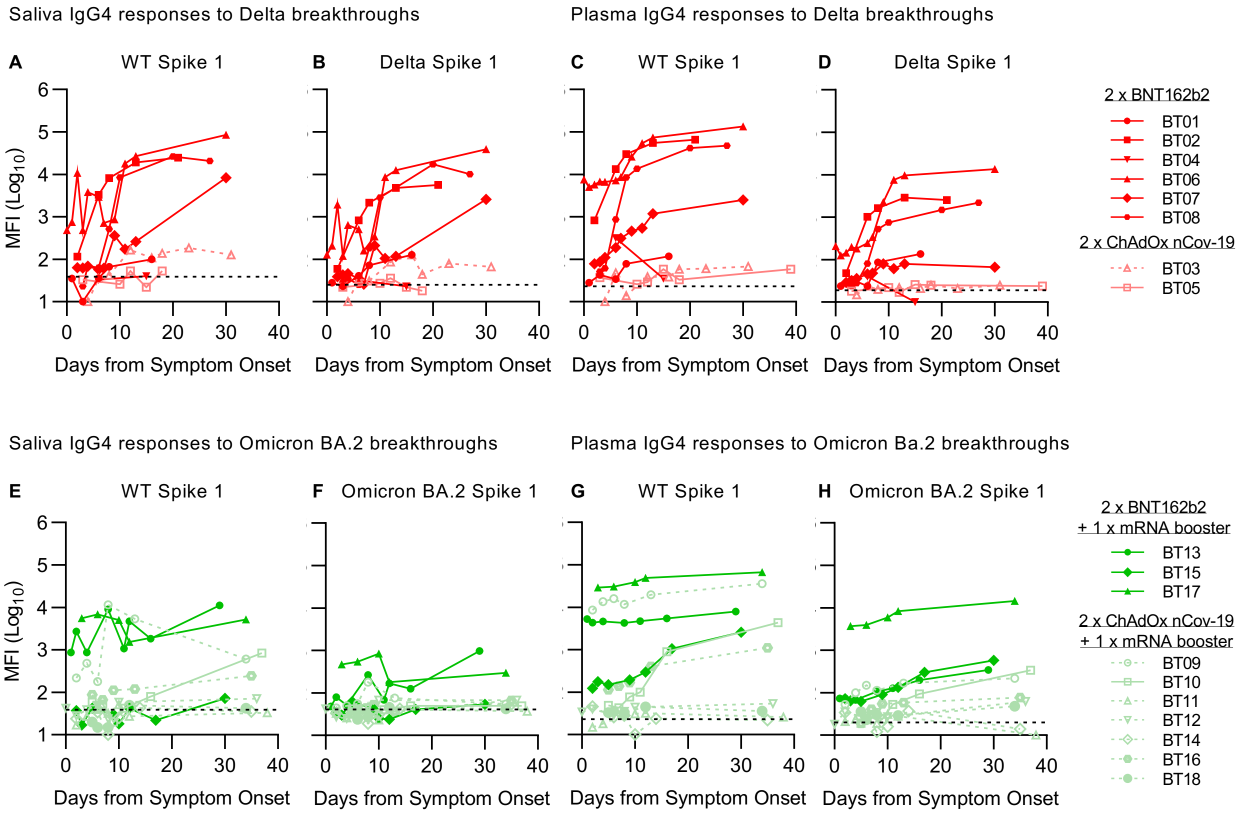

Supplementary figure 10: IgG4 anti-Spike 1 responses following breakthrough infection. Line graphs describe the salivary **(A, B, E and F)** and plasma **(C, D, G and H)** IgG4 responses post symptom onset against ancestral wildtype (WT) SARS-CoV-2 **(A, C, E and G)**, Delta **(B and D)** or Omicron BA.2 Spike 1 **(F and H)** respectively after Delta (*n* = 8) **(A-D)** and Omicron BA.2 (*n* = 10) **(E-H)** breakthrough infections. Solid lines depict individuals who received multiple mRNA vaccines (2 x BNT162b2; 2 x BNT162b2 + 1 x mRNA booster), while dotted lines depict individuals who did not receive mRNA vaccines (2 x ChAdOx nCov-19) or only received a single mRNA booster (2 x ChAdOx nCov-19 + 1 x mRNA booster).

Plasma SARS-CoV-2 VoC antibody signatures following breakthrough infections

% ACE2 Inhibition (≤5 days vs 2 weeks)

FcγR3a Responses (≤5 days vs 2 weeks)

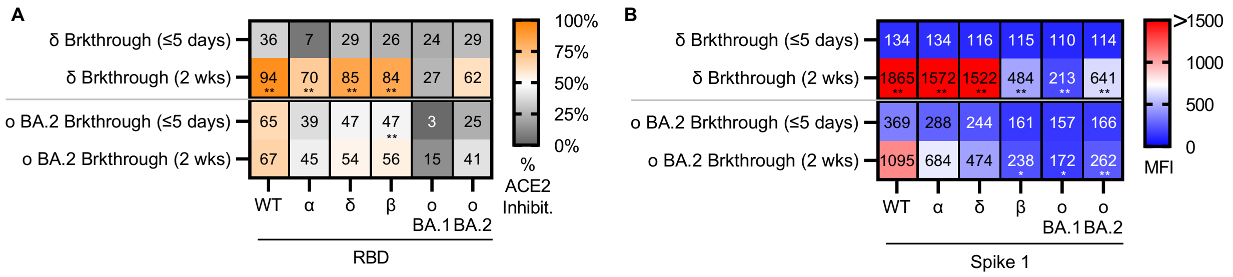

Supplementary Figure 11: Plasma antibody signatures following breakthrough infections. Heat maps compare the plasma inhibition of RBD-ACE2 interactions against the ancestral wildtype (WT) SARS-CoV-2 or the VoCs (α, Alpha; δ, Delta; β, Beta; o BA.1, Omicron BA.1; o BA.2, Omicron BA.2) between the start and clearance of Delta (*n* = 8) and Omicron BA.2 (*n* = 10) COVID-19 breakthrough infections **(A)**. Heat maps also describe the plasma antibody responses capable of FcγR3a engagement that are elicited early and 2 weeks post-symptom onset of Delta (*n* = 8) and Omicron BA.2 (*n* = 10) COVID-19 breakthrough infections **(B)**. Significant differences between both timepoints were calculated using Friedman's test followed by Dunn's test for multiple comparisons. Where significant or trending significance, *P*-values were reported (**P* ≤ 0.05; ***P* ≤ 0.01; ****P* ≤ 0.001; *****P* ≤ 0.0001).

Saliva antibody responses over time, breakthrough infections

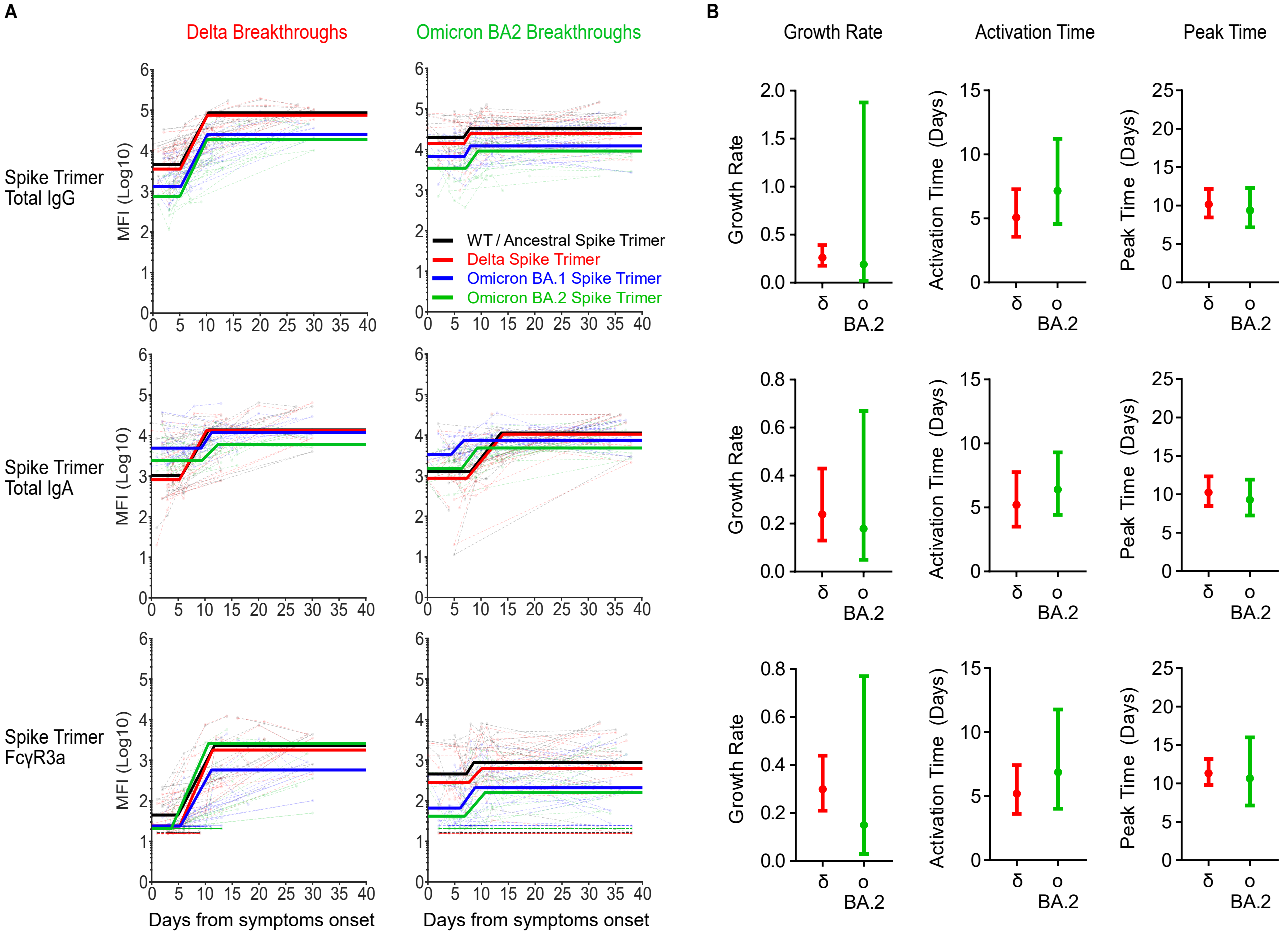

Supplementary Figure 12: Pre-existing vaccine-induced immunity modulate salivary antibody kinetics against Spike trimer during COVID-19 breakthrough infections. Modelled kinetic curves (WT: black; Delta: red; Omicron BA.1: blue; Omicron BA.2: green) describe the ancestral wildtype and variant-specific Spike Trimer antibody responses from serially collected saliva samples during Delta (*n* = 8) or Omicron BA.2 (*n* = 10) breakthrough infection for up to 40 days post-symptom onset **(A)**. Connected dotted lines indicate serial samples from the same individual. Lines with open circles at the bottom of each graph reflect samples that were excluded from the model for being below the threshold of detection (2 S.D. background readings). Dot plots displaying 95% confidence intervals beside each row of kinetic curves list the calculated growth rate, time to activation and time to peak of variant-specific salivary responses (Delta: red; Omicron BA.2: green) by their respective breakthrough cohorts (eg: Delta variant responses during the Delta breakthroughs) **(B)**.

Plasma antibody responses over time, breakthrough infections

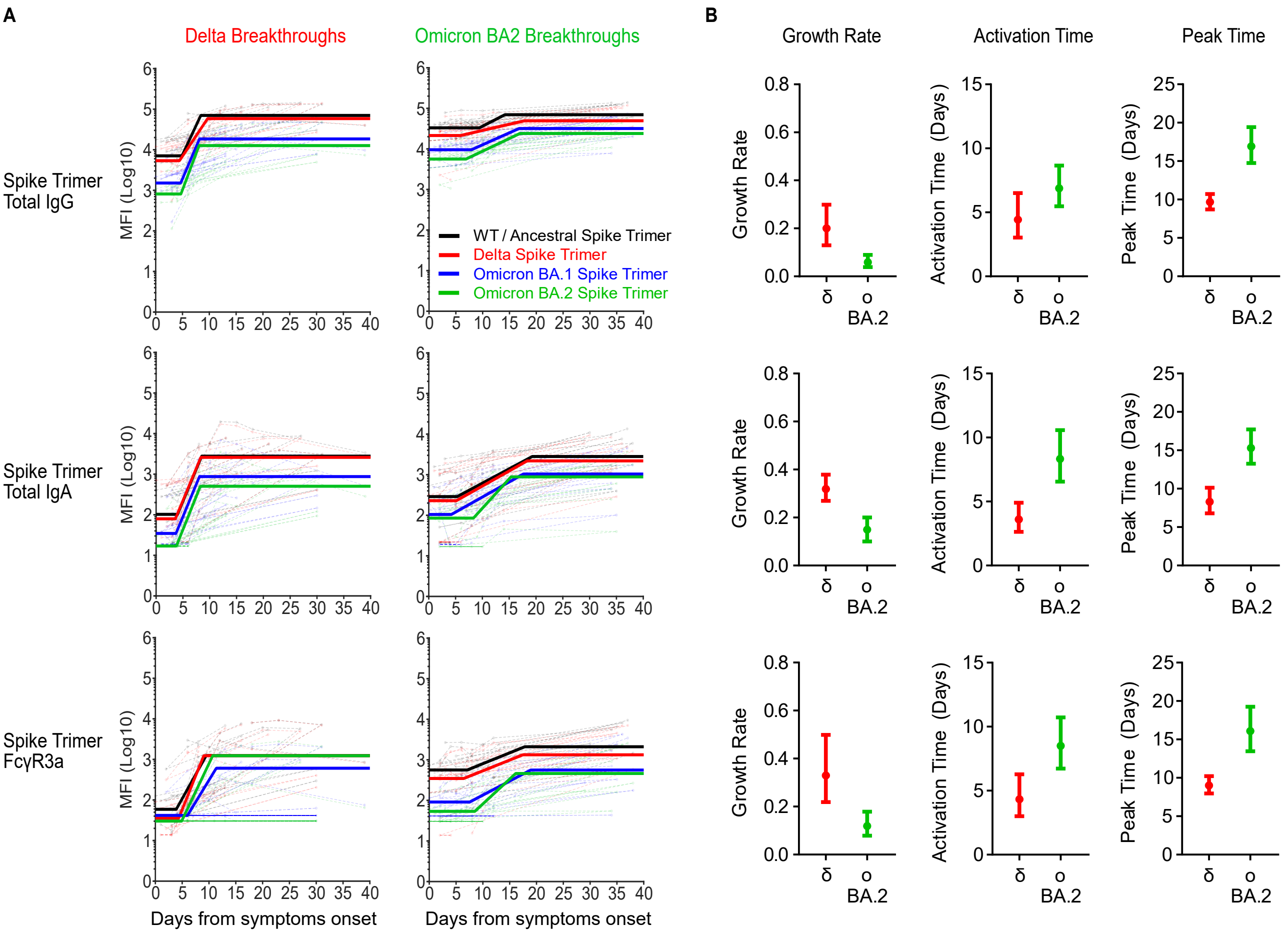

Supplementary Figure 13: Plasma antibody responses show delayed kinetics during Omicron BA.2 breakthrough infections. Modelled kinetic curves (WT: black; Delta: red; Omicron BA.1: blue; Omicron BA.2: green) describe the ancestral wildtype and variant-specific Spike Trimer antibody responses from serially collected plasma samples during Delta (*n* = 8) or Omicron BA.2 (*n* = 10) breakthrough infection for up to 40 days post-symptom onset **(A)**. Connected dotted lines indicate serial samples from the same individual. Lines with open circles at the bottom of each graph reflect samples that were excluded from the model for being below the threshold of detection (2 S.D. background readings). Dot plots displaying 95% confidence intervals beside each row of kinetic curves list the calculated growth rate, time to activation and time to peak of variant-specific plasma responses (Delta: red; Omicron BA.2: green) by their respective breakthrough cohorts (eg: Delta variant responses during the Delta breakthroughs) **(B)**.

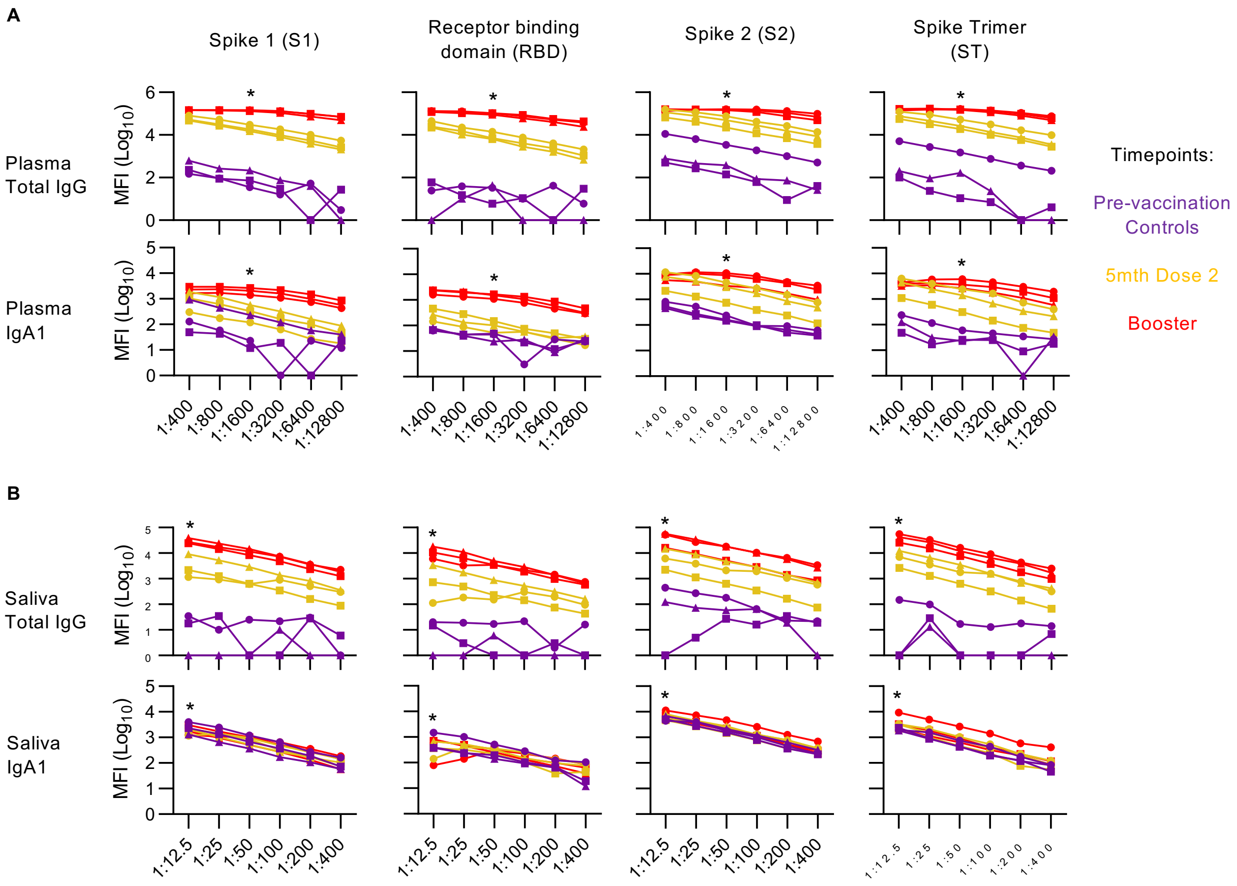

Supplementary Figure 14: Titration curves for SARS-CoV-2 ancestral Spike coupled arrays. Titration curves depict the plasma **(A)** and salivary **(B)** total IgG and IgA responses against ancestral wildtype (WT) Spike antigens using samples from three vaccinated individuals at the respective timepoints. Asterisks define the respective dilutions chosen for the multiplex assays.

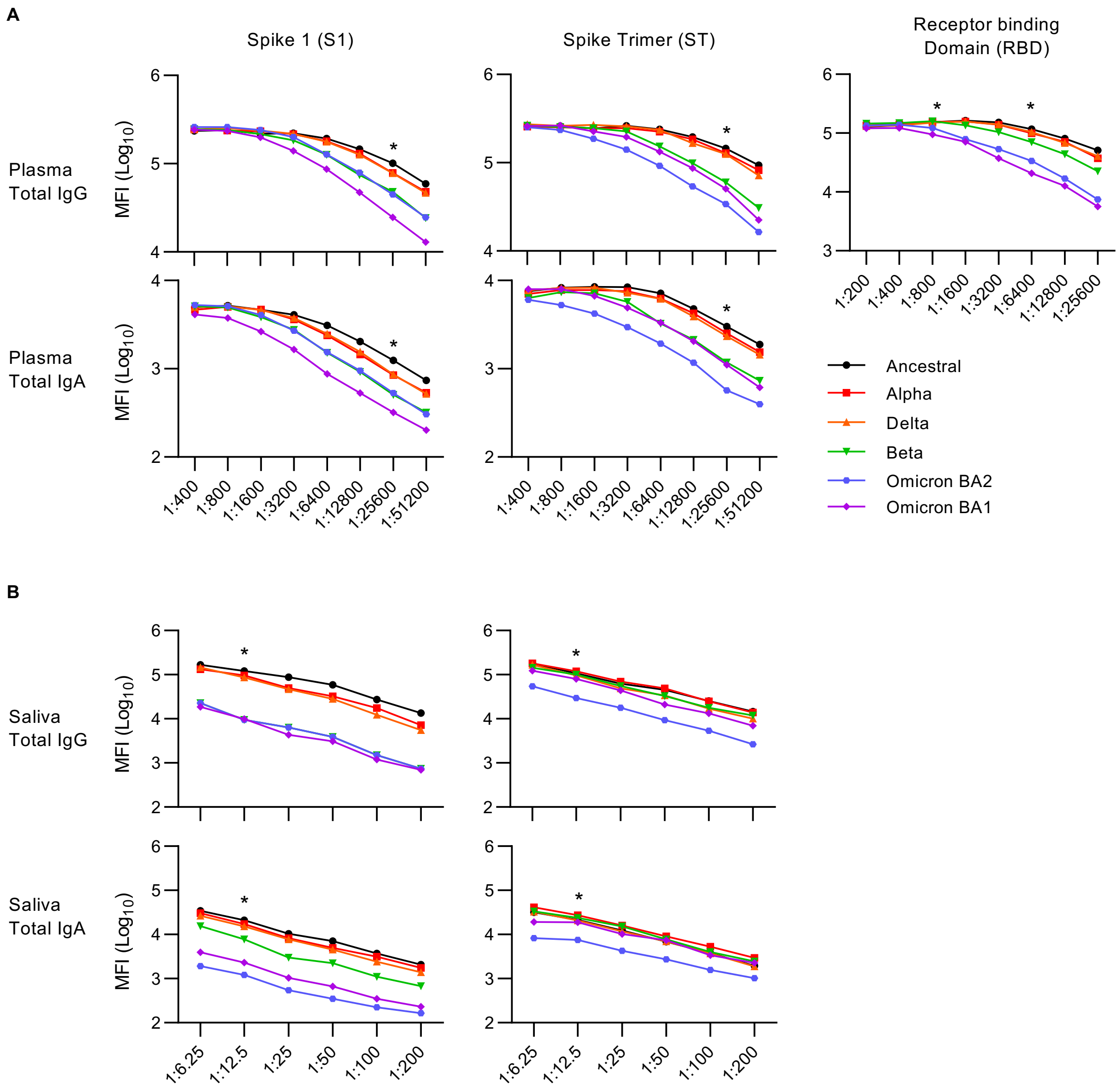

Supplementary Figure 15: Titration curves for SARS-CoV-2 variant Spike coupled arrays. Titration curves depict the plasma **(A)** and salivary **(B)** total IgG and IgA responses against ancestral wildtype (WT) Spike and the VoC Spikes using pooled plasma from COVID-19 boostered individuals and the saliva sample from a boostered individual who seroconverted strongly during their Delta breakthrough infection. Asterisks define the respective dilutions chosen for the multiplex assays.

Supplementary Table 1: Cohort information for COVID-19 vaccinees (V) and uninfected, unvaccinated controls (P)

Donor:	Gender:	Age:	Time from previous COVID-19 (days):	1st vaccine dose:	Timepoint post 1st dose (days):	Time between 1st and 2nd dose:	2nd vaccine dose:	Timepoint post 2nd dose (days):	Extended timepoint post 2nd dose (days)	3rd vaccine dose:	Timepoint post 3rd dose (days):
V01	F	In their 50s	n.d.	BNT162b2	n.d.	22	BNT162b2	12	224	BNT162b2	11
V02	M	In their 30s	n.d.	BNT162b2	11	23	BNT162b2	13	225	mRNA-1273	11
V03	F	In their 20s	n.d.	BNT162b2	10	22	BNT162b2	13	n.d.	n.d.	n.d.
V04	F	In their 20s	n.d.	BNT162b2	9	21	BNT162b2	13	225	BNT162b2	14
V05	M	In their 30s	n.d.	BNT162b2	n.d.	27	BNT162b2	14	219	BNT162b2	11
V06	F	In their 30s	n.d.	BNT162b2	9	28	BNT162b2	13	n.d.	n.d.	n.d.
V07	F	In their 30s	n.d.	BNT162b2	8	21	BNT162b2	12	224	BNT162b2	13
V08	F	In their 30s	n.d.	BNT162b2	n.d.	29	BNT162b2	13	217	BNT162b2	15
V09	F	In their 20s	n.d.	BNT162b2	10	24	BNT162b2	18	223	BNT162b2	10
V10	F	In their 20s	n.d.	BNT162b2	n.d.	33	BNT162b2	14	213	BNT162b2	14
V11	M	In their 30s	n.d.	BNT162b2	10	22	BNT162b2	13	225	BNT162b2	10
V12	F	In their 30s	n.d.	BNT162b2	10	22	BNT162b2	13	225	BNT162b2	13
V13	M	In their 30s	n.d.	BNT162b2	6	23	BNT162b2	15	n.d.	n.d.	n.d.
V14	F	In their 30s	n.d.	BNT162b2	6	23	BNT162b2	15	220	BNT162b2	14
V15	M	In their 30s	n.d.	BNT162b2	n.d.	21	BNT162b2	13	225	BNT162b2	13
V16	F	In their 50s	n.d.	BNT162b2	7	21	BNT162b2	13	225	mRNA-1273	11
V17	M	In their 30s	n.d.	BNT162b2	10	21	BNT162b2	14	226	BNT162b2	13
V18	M	In their 40s	n.d.	BNT162b2	12	33	BNT162b2	22	n.d.	n.d.	n.d.
V19	F	In their 30s	n.d.	BNT162b2	n.d.	23	BNT162b2	n.d.	197	BNT162b2	15
V20	F	In their 30s	n.d.	BNT162b2	n.d.	21	BNT162b2	n.d.	188	BNT162b2	10
V21	F	In their 20s	n.d.	ChAdOx nCoV-19	15	85	ChAdOx nCoV-19	14	168	mRNA-1273	14
V22	F	In their 50s	n.d.	ChAdOx nCoV-19	13	85	ChAdOx nCoV-19	12	n.d.	n.d.	n.d.
V23	M	In their 50s	n.d.	ChAdOx nCoV-19	13	80	ChAdOx nCoV-19	17	n.d.	n.d.	n.d.
V24	F	In their 60s	n.d.	ChAdOx nCoV-19	22	84	ChAdOx nCoV-19	14	n.d.	n.d.	n.d.
V25	M	In their 60s	n.d.	ChAdOx nCoV-19	14	70	ChAdOx nCoV-19	7	n.d.	n.d.	n.d.

V26	M	In their 60s	n.d.	ChAdOx nCoV-19	11	84	ChAdOx nCoV-19	10	n.d.	n.d.	n.d.
V27	M	In their 20s	n.d.	ChAdOx nCoV-19	15	71	ChAdOx nCoV-19	22	n.d.	n.d.	n.d.
V28	M	In their 50s	n.d.	ChAdOx nCoV-19	13	81	ChAdOx nCoV-19	n.d.	n.d.	n.d.	n.d.
V29	M	In their 60s	n.d.	ChAdOx nCoV-19	7	84	ChAdOx nCoV-19	7	n.d.	n.d.	n.d.
V30	M	In their 50s	n.d.	ChAdOx nCoV-19	16	84	ChAdOx nCoV-19	16	n.d.	n.d.	n.d.
V31	F	In their 50s	n.d.	ChAdOx nCoV-19	8	84	ChAdOx nCoV-19	15	n.d.	n.d.	n.d.
V32	F	In their 30s	n.d.	ChAdOx nCoV-19	n.d.	81	ChAdOx nCoV-19	n.d.	101	BNT162b2	14
V33	F	In their 20s	n.d.	ChAdOx nCoV-19	n.d.	84	ChAdOx nCoV-19	n.d.	124	BNT162b2	10
V34	F	In their 20s	n.d.	ChAdOx nCoV-19	n.d.	84	ChAdOx nCoV-19	n.d.	134	BNT162b2	14
V35	F	In their 50s	n.d.	ChAdOx nCoV-19	n.d.	91	ChAdOx nCoV-19	n.d.	158	BNT162b2	29
V36	F	In their 30s	n.d.	ChAdOx nCoV-19	n.d.	90	ChAdOx nCoV-19	n.d.	178	BNT162b2	29
V37	F	In their 20s	n.d.	ChAdOx nCoV-19	n.d.	92	ChAdOx nCoV-19	n.d.	184	BNT162b2	31
V38	F	In their 50s	501	BNT162b2	18	22	BNT162b2	19	n.d.	n.d.	n.d.
V39	M	In their 60s	412	BNT162b2	7	48	BNT162b2	22	n.d.	n.d.	n.d.
V40	F	In their 20s	534	BNT162b2	7	25	BNT162b2	19	n.d.	n.d.	n.d.
V41	M	In their 50s	483	BNT162b2	12	31	BNT162b2	16	n.d.	n.d.	n.d.
V42	F	In their 60s	479	BNT162b2	12	21	BNT162b2	13	n.d.	n.d.	n.d.
V43	M	In their 60s	493	BNT162b2	13	21	BNT162b2	34	n.d.	n.d.	n.d.
V44	F	In their 50s	493	BNT162b2	13	21	BNT162b2	34	n.d.	n.d.	n.d.
V45	F	In their 20s	431	BNT162b2	13	25	BNT162b2	13	n.d.	n.d.	n.d.
V46	M	In their 50s	438	BNT162b2	14	21	BNT162b2	10	n.d.	n.d.	n.d.
V47	F	In their 30s	517	BNT162b2	16	42	BNT162b2	21	n.d.	n.d.	n.d.
P01	F	In their 30s	n.d.	n.d.	n.d.	n.d.	n.d.	n.d.	n.d.	n.d.	n.d.
P02	F	In their 20s	n.d.	n.d.	n.d.	n.d.	n.d.	n.d.	n.d.	n.d.	n.d.
P03	F	In their 30s	n.d.	n.d.	n.d.	n.d.	n.d.	n.d.	n.d.	n.d.	n.d.
P04	M	In their 20s	n.d.	n.d.	n.d.	n.d.	n.d.	n.d.	n.d.	n.d.	n.d.
P05	F	In their 30s	n.d.	n.d.	n.d.	n.d.	n.d.	n.d.	n.d.	n.d.	n.d.
P06	F	In their 20s	n.d.	n.d.	n.d.	n.d.	n.d.	n.d.	n.d.	n.d.	n.d.
P07	F	In their 20s	n.d.	n.d.	n.d.	n.d.	n.d.	n.d.	n.d.	n.d.	n.d.
P08	F	In their 20s	n.d.	n.d.	n.d.	n.d.	n.d.	n.d.	n.d.	n.d.	n.d.

P09	F	In their 30s	n.d.	n.d.	n.d.	n.d.	n.d.	n.d.	n.d.	n.d.	n.d.
P10	F	In their 30s	n.d.	n.d.	n.d.	n.d.	n.d.	n.d.	n.d.	n.d.	n.d.
P11	M	In their 30s	n.d.	n.d.	n.d.	n.d.	n.d.	n.d.	n.d.	n.d.	n.d.
P12	F	In their 20s	n.d.	n.d.	n.d.	n.d.	n.d.	n.d.	n.d.	n.d.	n.d.
P13	M	In their 30s	n.d.	n.d.	n.d.	n.d.	n.d.	n.d.	n.d.	n.d.	n.d.
P14	F	In their 30s	n.d.	n.d.	n.d.	n.d.	n.d.	n.d.	n.d.	n.d.	n.d.
P15	F	In their 30s	n.d.	n.d.	n.d.	n.d.	n.d.	n.d.	n.d.	n.d.	n.d.
P16	F	In their 20s	n.d.	n.d.	n.d.	n.d.	n.d.	n.d.	n.d.	n.d.	n.d.
P17	F	In their 20s	n.d.	n.d.	n.d.	n.d.	n.d.	n.d.	n.d.	n.d.	n.d.
P18	M	In their 30s	n.d.	n.d.	n.d.	n.d.	n.d.	n.d.	n.d.	n.d.	n.d.
P19	F	In their 60s	n.d.	n.d.	n.d.	n.d.	n.d.	n.d.	n.d.	n.d.	n.d.
P20	F	In their 20s	n.d.	n.d.	n.d.	n.d.	n.d.	n.d.	n.d.	n.d.	n.d.

Supplementary Table 2. Cohort of SARS-CoV-2 breakthrough infections

Donor:	Age:	Gender:	Vaccination History:	Time from last vaccination till breakthrough infection (months):	Timepoints post onset for plasma / saliva samples (bold: incl. nasal swabs):	Breakthrough VoC
BT01	In their 50s	F	2 x BNT162b2	5.3	1, **3, 6,** 8, 16	Delta
BT02	In their 30s	M	2 x BNT162b2	4.3	**2, 6,** 8, 13, 21	Delta
BT03	In their 50s	F	2 x ChAdOx nCoV-19	3	**4, 6, 8, 12,** 16, 18, 23, 31	Delta
BT04	In their teens	F	2 x BNT162b2	0.3	6, **15**	Delta
BT05	In their 20s	M	2 x ChAdOx nCoV-19	1	3, 10, **12,** 15, 18, 39	Delta
BT06	In their 30s	F	2 x BNT162b2	4.6	0, **1, 2, 3, 4, 6, 7, 9,** 11, 13, 30	Delta
BT07	In their 30s	F	2 x BNT162b2	3.6	**2, 3, 4, 6, 7, 9 ,11, 13**, 30	Delta
BT08	In their 20s	M	2 x BNT162b2	4.3	**3, 4, 6, 8,** 10, 20, 27	Delta
BT09	In their 50s	F	2 x ChAdOx nCoV-19<br>1 x mRNA-1273	4.3	**2, 4, 6, 8,** 13, 34	Omicron BA.2
BT10	In their 50s	M	2 x ChAdOx nCoV-19<br>1 x mRNA-1273	4	**5, 7, 9, 11,** 16, 37	Omicron BA.2
BT11	In their 50s	F	2 x ChAdOx nCoV-19<br>1 x mRNA-1273	5.3	2, **4, 8, 10**, 12, 38	Omicron BA.2
BT12	In their 60s	M	2 x ChAdOx nCoV-19<br>1 x mRNA-1273	2.6	0, **2, 6, 8,** 10, 36	Omicron BA.2
BT13	In their 40s	F	3 x BNT162b2	4	**1, 2, 4**, 8, **11,** 16, 29	Omicron BA.2
BT14	In their 50s	F	2 x ChAdOx nCoV-19<br>1 x mRNA-1273	2	**2,** 6, 8, **10**, 14, 35	Omicron BA.2
BT15	In their 40s	M	3 x BNT162b2	1.6	**2, 3,** 5, 9, **12**, 17, 30	Omicron BA.2
BT16	In their 60s	M	2 x ChAdOx nCoV-19<br>1 x mRNA-1273	3.3	**5,** 7, **9,** 13, 35	Omicron BA.2
BT17	In their 40s	F	2 x BNT162b2<br>1 x mRNA-1273	3	**3, 6,** 10, 12, 34	Omicron BA.2
BT18	In their 60s	M	2 x ChAdOx nCoV-19<br>1 x BNT162b2	1.6	**5**, 6, **8,** 12, 34	Omicron BA.2

Supplementary Table 3: Parameters and confidence intervals for modelled kinetic curves of COVID-19 breakthrough cohorts

Delta breakthrough cohort (Plasma)

**Sample: Plasma Detector: Pan IgG**	**WT Spike 1**			**Delta Spike 1**			**BA1 Spike 1**			**BA2 Spike 1**		
	Lower CI	Mean	Upper CI	Lower CI	Mean	Upper CI	Lower CI	Mean	Upper CI	Lower CI	Mean	Upper CI
Initial MFI:	2452.00	4570.88	8520.79	1450.37	2691.53	4994.82	147.64	416.87	1177.06	474.68	977.24	2011.87
Growth rate:	0.18	0.26	0.37	0.24	0.32	0.43	0.35	0.49	0.68	0.35	0.46	0.60
Activation time (days):	3.25	4.31	5.70	3.37	4.26	5.39	3.72	4.48	5.40	3.67	4.44	5.37
Peak time (days):	7.35	8.58	10.03	7.08	8.17	9.42	6.40	7.39	8.54	6.46	7.39	8.45
Maximum MFI:	60022.75	80786.00	108731.74	40682.38	58140.08	83089.26	7549.81	12057.31	19255.94	15847.52	25655.55	41533.76
**Sample: Plasma Detector: Pan IgG**	**WT Spike Trimer**			**Delta Spike Trimer**			**BA1 Spike Trimer**			**BA2 Spike Trimer**		
	Lower CI	Mean	Upper CI	Lower CI	Mean	Upper CI	Lower CI	Mean	Upper CI	Lower CI	Mean	Upper CI
Initial MFI:	3533.13	7079.46	14185.35	2340.78	5370.32	12320.83	731.88	1513.56	3130.11	426.30	812.83	1549.82
Growth rate:	0.22	0.28	0.37	0.13	0.20	0.30	0.24	0.31	0.41	0.27	0.35	0.47
Activation time (days):	3.79	4.90	6.34	3.03	4.44	6.50	3.69	4.66	5.90	3.79	4.71	5.86
Peak time (days):	7.27	8.41	9.74	8.74	9.68	10.72	7.05	8.17	9.46	7.04	8.08	9.28
Maximum MFI:	63778.05	87397.54	119764.24	52597.18	75669.39	108862.42	19256.18	29932.47	46528.08	11581.74	19694.09	33488.68
**Sample: Plasma Detector: IgA**	**WT Spike 1**			**Delta Spike 1**			**BA1 Spike 1**			**BA2 Spike 1**		
	Lower CI	Mean	Upper CI	Lower CI	Mean	Upper CI	Lower CI	Mean	Upper CI	Lower CI	Mean	Upper CI
Initial MFI:	29.96	50.12	83.84	11.71	19.95	33.98	10.47	10.47	10.47	14.45	14.45	14.45
Growth rate:	0.26	0.31	0.36	0.28	0.46	0.76	0.21	0.48	1.09	0.17	0.55	1.80
Activation time (days):	2.69	4.06	6.12	3.08	4.18	5.66	3.77	4.85	6.25	2.60	3.82	5.61
Peak time (days):	7.50	8.94	10.65	6.51	7.69	9.09	6.44	7.32	8.31	5.69	7.17	9.04
Maximum MFI:	1139.05	3602.69	11394.95	731.38	2240.54	6863.79	59.48	202.24	687.63	144.51	506.60	1776.00
**Sample: Plasma Detector: IgA**	**WT Spike Trimer**			**Delta Spike Trimer**			**BA1 Spike Trimer**			**BA2 Spike Trimer**		
	Lower CI	Mean	Upper CI	Lower CI	Mean	Upper CI	Lower CI	Mean	Upper CI	Lower CI	Mean	Upper CI
Initial MFI:	40.39	102.33	259.27	27.75	79.43	227.34	17.38	34.67	69.16	16.98	16.98	16.98
Growth rate:	0.26	0.32	0.40	0.27	0.32	0.38	0.21	0.32	0.48	0.19	0.33	0.57
Activation time (days):	2.97	4.01	5.43	2.63	3.60	4.91	2.52	3.71	5.44	2.51	3.78	5.70
Peak time (days):	7.08	8.50	10.21	6.83	8.33	10.15	6.93	8.08	9.44	7.06	8.25	9.64
Maximum MFI:	2715.44	6904.71	17557.04	2572.90	6526.89	16557.30	784.21	2646.87	8933.70	422.03	1412.80	4729.51
**Sample: Plasma Detector: FcγR3a**	**WT Spike 1**			**Delta Spike 1**			**BA1 Spike 1**			**BA2 Spike 1**		
	Lower CI	Mean	Upper CI	Lower CI	Mean	Upper CI	Lower CI	Mean	Upper CI	Lower CI	Mean	Upper CI
Initial MFI:	10.58	22.39	47.35	7.73	7.73	7.73	7.00	7.00	7.00	12.02	12.02	12.02
Growth rate:	0.29	0.41	0.58	0.39	0.63	1.02	0.29	0.71	1.74	0.32	0.72	1.59
Activation time (days):	3.04	4.14	5.63	3.18	4.26	5.72	4.23	5.16	6.28	3.69	4.62	5.77
Peak time (days):	7.43	8.58	9.92	6.88	7.85	8.95	6.13	6.89	7.74	6.13	6.89	7.75
Maximum MFI:	1149.22	2440.14	5181.15	500.78	1307.45	3413.48	34.33	81.56	193.77	80.78	289.76	1039.33
**Sample: Plasma Detector: FcγR3a**	**WT Spike Trimer**			**Delta Spike Trimer**			**BA1 Spike Trimer**			**BA2 Spike Trimer**		
	Lower CI	Mean	Upper CI	Lower CI	Mean	Upper CI	Lower CI	Mean	Upper CI	Lower CI	Mean	Upper CI
Initial MFI:	19.93	58.88	173.94	15.26	35.48	82.51	41.69	41.69	41.69	30.20	30.20	30.20
Growth rate:	0.14	0.24	0.39	0.22	0.33	0.50	0.12	0.22	0.40	0.17	0.28	0.46
Activation time (days):	3.06	3.90	4.97	3.01	4.35	6.29	4.99	5.99	7.19	4.11	4.90	5.85
Peak time (days):	7.97	9.49	11.29	7.96	9.03	10.23	9.77	11.36	13.21	9.28	10.70	12.34
Maximum MFI:	1280.79	2880.93	6480.22	889.36	2214.45	5513.83	206.32	569.38	1571.34	116.46	360.96	1118.75

Delta breakthrough cohort (Saliva)

Sample: Saliva<br>Detector: Pan IgG	WT Spike 1			Delta Spike 1			BA1 Spike 1			BA2 Spike 1		
	Lower CI	Mean	Upper CI	Lower CI	Mean	Upper CI	Lower CI	Mean	Upper CI	Lower CI	Mean	Upper CI
Initial MFI:	1456.26	2630.27	4750.73	757.74	1412.54	2633.18	83.96	208.93	519.90	196.01	398.11	808.57
Growth rate:	0.23	0.28	0.33	0.15	0.32	0.67	0.26	0.36	0.51	0.18	0.40	0.90
Activation time (days):	3.24	4.81	7.14	3.21	4.81	7.20	2.03	4.18	8.61	2.03	4.39	9.51
Peak time (days):	8.61	10.07	11.79	6.98	9.87	13.97	7.72	9.12	10.77	7.36	8.94	10.85
Maximum MFI:	52419.76	82960.99	131296.40	34349.48	59621.86	103488.23	8189.38	15292.07	28554.98	15572.19	29617.63	56331.43

Sample: Saliva<br>Detector: Pan IgG	WT Spike Trimer			Delta Spike Trimer			BA1 Spike Trimer			BA2 Spike Trimer		
	Lower CI	Mean	Upper CI	Lower CI	Mean	Upper CI	Lower CI	Mean	Upper CI	Lower CI	Mean	Upper CI
Initial MFI:	2333.24	4570.88	8954.47	1778.77	3548.13	7077.50	791.63	1318.26	2195.23	453.48	758.58	1268.94
Growth rate:	0.16	0.24	0.37	0.18	0.26	0.39	0.18	0.26	0.38	0.22	0.28	0.35
Activation time (days):	3.56	5.10	7.32	3.58	5.10	7.28	3.58	5.21	7.57	3.74	5.05	6.82
Peak time (days):	8.32	10.38	12.95	8.49	10.18	12.19	8.30	10.18	12.48	8.74	10.07	11.61
Maximum MFI:	59092.74	83926.20	119195.80	48672.35	72419.92	107754.08	19554.12	31559.85	50936.77	11795.82	21236.87	38234.29

Sample: Saliva<br>Detector: IgA	WT Spike 1			Delta Spike 1			BA1 Spike 1			BA2 Spike 1		
	Lower CI	Mean	Upper CI	Lower CI	Mean	Upper CI	Lower CI	Mean	Upper CI	Lower CI	Mean	Upper CI
Initial MFI:	1277.50	2089.30	3416.96	586.81	870.96	1292.71	12.02	12.02	12.02	7.80	19.05	46.57
Growth rate:	0.18	0.32	0.56	0.13	0.28	0.60	0.21	0.32	0.49	0.20	0.35	0.62
Activation time (days):	6.41	7.39	8.52	5.64	7.10	8.93	4.55	6.05	8.04	3.78	5.58	8.25
Peak time (days):	8.50	9.49	10.59	8.76	9.97	11.35	8.27	9.12	10.05	8.50	10.38	12.68
Maximum MFI:	12793.77	24412.07	46581.21	24012.78	37021.41	57077.32	5170.17	8866.42	15205.17	6382.85	11109.55	19336.50

Sample: Saliva<br>Detector: IgA	WT Spike Trimer			Delta Spike Trimer			BA1 Spike Trimer			BA2 Spike Trimer		
	Lower CI	Mean	Upper CI	Lower CI	Mean	Upper CI	Lower CI	Mean	Upper CI	Lower CI	Mean	Upper CI
Initial MFI:	405.70	1023.29	2581.07	354.29	812.83	1864.83	2699.48	4897.79	8886.28	1587.32	2454.71	3796.09
Growth rate:	0.13	0.22	0.39	0.13	0.24	0.43	0.08	0.20	0.47	0.02	0.13	0.99
Activation time (days):	3.77	5.64	8.43	3.50	5.21	7.75	7.86	9.30	11.00	6.89	9.49	13.06
Peak time (days):	9.03	10.70	12.67	8.54	10.28	12.37	9.55	11.25	13.24	8.96	12.43	17.24
Maximum MFI:	10434.39	26618.42	67904.31	44129.44	60879.45	83987.18	12923.19	22500.64	39176.01	6465.54	12044.92	22438.96

Sample: Saliva<br>Detector: FcγR3a	WT Spike 1			Delta Spike 1			BA1 Spike 1			BA2 Spike 1		
	Lower CI	Mean	Upper CI	Lower CI	Mean	Upper CI	Lower CI	Mean	Upper CI	Lower CI	Mean	Upper CI
Initial MFI:	6.03	15.49	39.78	18.20	18.20	18.20	16.98	16.98	16.98	11.75	11.75	11.75
Growth rate:	0.26	0.39	0.60	0.22	0.35	0.54	0.12	0.23	0.47	0.27	0.40	0.59
Activation time (days):	2.94	4.53	6.98	3.66	5.10	7.11	4.72	6.05	7.76	4.57	5.81	7.40
Peak time (days):	8.51	10.07	11.92	9.43	10.91	12.63	6.88	8.50	10.50	7.67	9.03	10.61
Maximum MFI:	1045.81	2594.15	6434.81	446.74	1508.93	5096.59	26.45	111.68	471.51	79.56	394.54	1956.52

Sample: Saliva<br>Detector: FcγR3a	WT Spike Trimer			Delta Spike Trimer			BA1 Spike Trimer			BA2 Spike Trimer		
	Lower CI	Mean	Upper CI	Lower CI	Mean	Upper CI	Lower CI	Mean	Upper CI	Lower CI	Mean	Upper CI
Initial MFI:	18.53	44.67	107.70	11.60	23.99	49.61	23.99	23.99	23.99	20.89	20.89	20.89
Growth rate:	0.16	0.25	0.39	0.21	0.30	0.44	0.13	0.24	0.44	0.17	0.30	0.52
Activation time (days):	3.11	4.90	7.73	3.64	5.21	7.44	3.72	5.37	7.74	2.15	3.63	6.13
Peak time (days):	9.80	11.70	13.97	9.80	11.36	13.17	9.12	11.13	13.60	8.62	10.59	13.01
Maximum MFI:	1705.03	3455.75	7004.10	1193.14	2761.96	6393.56	230.85	707.85	2170.46	146.19	490.87	1648.24

Omicron BA.2 breakthrough cohort (Plasma)

Sample: Plasma Detector: Pan IgG	WT Spike 1			Delta Spike 1			BA1 Spike 1			BA2 Spike 1		
	Lower CI	Mean	Upper CI	Lower CI	Mean	Upper CI	Lower CI	Mean	Upper CI	Lower CI	Mean	Upper CI
Initial MFI:	13688.00	19952.62	29084.39	6957.29	11481.54	18947.85	921.38	1905.46	3940.58	2505.65	4365.16	7604.66
Growth rate:	0.04	0.06	0.08	0.05	0.07	0.10	0.14	0.29	0.58	0.05	0.07	0.11
Activation time (days):	6.89	8.41	10.28	6.30	7.92	9.97	6.57	7.39	8.32	4.87	6.17	7.82
Peak time (days):	14.68	16.78	19.17	12.81	14.59	16.61	8.06	8.76	9.51	11.89	14.01	16.52
Maximum MFI:	43394.17	64444.81	95707.19	24012.78	37021.41	57077.32	3585.41	5948.38	9868.69	6382.85	11109.55	19336.50

Sample: Plasma Detector: Pan IgG	WT Spike Trimer			Delta Spike Trimer			BA1 Spike Trimer			BA2 Spike Trimer		
	Lower CI	Mean	Upper CI	Lower CI	Mean	Upper CI	Lower CI	Mean	Upper CI	Lower CI	Mean	Upper CI
Initial MFI:	23722.26	33113.11	46221.50	12495.68	21379.62	36579.69	6604.93	9549.93	13808.04	3712.62	5623.41	8517.65
Growth rate:	0.04	0.07	0.13	0.02	0.03	0.05	0.04	0.06	0.08	0.04	0.06	0.09
Activation time (days):	7.81	9.39	11.30	4.49	5.87	7.68	6.46	7.92	9.72	5.49	6.89	8.65
Peak time (days):	12.57	14.15	15.94	14.84	17.81	21.38	14.57	16.78	19.32	14.78	16.95	19.43
Maximum MFI:	58368.56	79623.49	108618.41	44129.44	60879.45	83987.18	21860.87	34326.63	53900.75	17467.57	25411.93	36969.44

Sample: Plasma Detector: IgA	WT Spike 1			Delta Spike 1			BA1 Spike 1			BA2 Spike 1		
	Lower CI	Mean	Upper CI	Lower CI	Mean	Upper CI	Lower CI	Mean	Upper CI	Lower CI	Mean	Upper CI
Initial MFI:	68.30	141.25	292.12	21.08	40.74	78.73	10.47	10.47	10.47	14.45	14.45	14.45
Growth rate:	0.07	0.10	0.16	0.07	0.12	0.20	0.11	0.35	1.08	0.08	0.12	0.18
Activation time (days):	4.59	6.17	8.30	3.23	4.66	6.73	6.10	8.00	10.51	1.73	2.97	5.12
Peak time (days):	14.12	16.95	20.34	12.07	15.49	19.86	10.42	12.68	15.43	13.81	16.78	20.39
Maximum MFI:	1214.34	2706.92	6034.04	573.14	1200.10	2512.88	40.95	103.61	262.15	213.79	529.71	1312.47

Sample: Plasma Detector: IgA	WT Spike Trimer			Delta Spike Trimer			BA1 Spike Trimer			BA2 Spike Trimer		
	Lower CI	Mean	Upper CI	Lower CI	Mean	Upper CI	Lower CI	Mean	Upper CI	Lower CI	Mean	Upper CI
Initial MFI:	165.55	288.40	502.44	137.57	229.09	381.49	56.43	104.71	194.32	49.52	85.11	146.29
Growth rate:	0.04	0.07	0.12	0.04	0.07	0.13	0.05	0.07	0.11	0.10	0.15	0.20
Activation time (days):	3.88	5.31	7.27	3.65	5.16	7.28	2.59	4.22	6.88	6.55	8.33	10.60
Peak time (days):	14.93	19.30	24.95	14.17	18.36	23.78	14.27	17.64	21.79	13.25	15.33	17.74
Maximum MFI:	1876.28	4043.89	8715.69	1485.65	3058.28	6295.62	671.78	1487.06	3291.78	381.20	908.36	2164.53

Sample: Plasma Detector: FcγR3a	WT Spike 1			Delta Spike 1			BA1 Spike 1			BA2 Spike 1		
	Lower CI	Mean	Upper CI	Lower CI	Mean	Upper CI	Lower CI	Mean	Upper CI	Lower CI	Mean	Upper CI
Initial MFI:	106.65	208.93	409.30	45.39	79.43	139.01	7.00	7.00	7.00	8.52	14.13	23.42
Growth rate:	0.04	0.07	0.11	0.04	0.06	0.09	0.05	0.11	0.27	0.06	0.09	0.14
Activation time (days):	5.86	7.32	9.13	4.60	5.99	7.80	6.10	8.00	10.51	2.41	4.14	7.11
Peak time (days):	15.88	18.92	22.53	15.50	18.73	22.62	8.98	11.36	14.37	13.53	16.95	21.23
Maximum MFI:	728.37	1670.04	3829.10	258.17	629.14	1533.16	11.66	21.37	39.15	92.16	216.25	507.43

Sample: Plasma Detector: FcγR3a	WT Spike Trimer			Delta Spike Trimer			BA1 Spike Trimer			BA2 Spike Trimer		
	Lower CI	Mean	Upper CI	Lower CI	Mean	Upper CI	Lower CI	Mean	Upper CI	Lower CI	Mean	Upper CI
Initial MFI:	283.19	562.34	1116.66	149.10	346.74	806.34	62.96	91.20	132.10	36.94	53.70	78.07
Growth rate:	0.03	0.05	0.09	0.03	0.05	0.09	0.04	0.07	0.11	0.08	0.12	0.18
Activation time (days):	5.71	7.10	8.82	4.88	6.42	8.45	6.06	7.54	9.37	6.73	8.50	10.73
Peak time (days):	14.89	17.81	21.32	14.45	17.46	21.11	15.59	18.92	22.95	13.47	16.12	19.29
Maximum MFI:	1514.36	2750.59	4996.00	974.73	1835.16	3455.11	279.84	713.45	1818.96	190.99	446.85	1045.46

Omicron BA.2 breakthrough cohort (Saliva)

Sample: Saliva<br>Detector: Pan IgG	WT Spike 1			Delta Spike 1			BA1 Spike 1			BA2 Spike 1		
	Lower CI	Mean	Upper CI	Lower CI	Mean	Upper CI	Lower CI	Mean	Upper CI	Lower CI	Mean	Upper CI
Initial MFI:	6322.37	10964.78	19016.04	2778.69	5370.32	10379.11	543.75	1000.00	1839.08	1040.78	1905.46	3488.51
Growth rate:	0.01	0.27	6.03	0.00	0.27	17.04	0.00	0.27	454.14	0.02	0.25	2.78
Activation time (days):	4.84	7.17	10.63	4.06	7.10	12.41	3.11	7.24	16.86	4.76	7.24	11.02
Peak time (days):	6.87	8.33	10.10	6.66	8.25	10.21	6.44	8.17	10.35	7.26	9.30	11.91
Maximum MFI:	26945.80	45975.98	78446.02	12113.07	24084.05	47885.57	2331.57	4606.06	9099.35	6490.79	12285.01	23251.65

Sample: Saliva<br>Detector: Pan IgG	WT Spike Trimer			Delta Spike Trimer			BA1 Spike Trimer			BA2 Spike Trimer		
	Lower CI	Mean	Upper CI	Lower CI	Mean	Upper CI	Lower CI	Mean	Upper CI	Lower CI	Mean	Upper CI
Initial MFI:	12478.43	19952.62	31903.62	7999.08	14125.38	24943.65	4326.69	6760.83	10564.38	1963.54	3467.37	6122.94
Growth rate:	0.02	0.19	2.02	0.00	0.22	1071.06	0.03	0.23	1.65	0.02	0.19	1.88
Activation time (days):	5.17	6.75	8.82	4.98	6.82	9.35	5.48	6.89	8.67	4.58	7.17	11.23
Peak time (days):	6.07	7.92	10.35	2.77	7.92	22.66	6.54	8.00	9.80	7.17	9.39	12.31
Maximum MFI:	39325.96	62988.29	100888.18	29573.63	50590.79	86544.28	17800.46	28637.84	46073.28	9207.57	17454.63	33088.45

Sample: Saliva<br>Detector: IgA	WT Spike 1			Delta Spike 1			BA1 Spike 1			BA2 Spike 1		
	Lower CI	Mean	Upper CI	Lower CI	Mean	Upper CI	Lower CI	Mean	Upper CI	Lower CI	Mean	Upper CI
Initial MFI:	1031.43	1905.46	3520.14	393.59	870.96	1927.35	12.02	12.02	12.02	1.31	5.42	22.46
Growth rate:	0.05	0.10	0.22	0.06	0.13	0.26	0.19	0.33	0.57	0.18	0.29	0.47
Activation time (days):	6.70	8.85	11.69	6.51	8.33	10.66	8.79	11.47	14.98	3.49	5.37	8.26
Peak time (days):	11.50	14.01	17.08	10.28	12.55	15.33	12.29	15.33	19.13	10.95	13.20	15.90
Maximum MFI:	5968.77	12172.47	24824.05	2921.63	6310.56	13630.49	151.31	310.02	635.21	745.41	2012.94	5435.84

Sample: Saliva<br>Detector: IgA	WT Spike Trimer			Delta Spike Trimer			BA1 Spike Trimer			BA2 Spike Trimer		
	Lower CI	Mean	Upper CI	Lower CI	Mean	Upper CI	Lower CI	Mean	Upper CI	Lower CI	Mean	Upper CI
Initial MFI:	666.56	1288.25	2489.77	417.37	870.96	1817.52	2197.05	3388.44	5225.89	1019.76	1513.56	2246.47
Growth rate:	0.09	0.17	0.31	0.10	0.16	0.25	0.05	0.15	0.46	0.05	0.18	0.67
Activation time (days):	6.06	8.08	10.78	5.63	7.39	9.70	2.88	4.44	6.83	4.43	6.42	9.32
Peak time (days):	11.60	13.74	16.26	11.75	14.15	17.05	4.90	6.82	9.50	7.25	9.30	11.93
Maximum MFI:	11746.97	18413.92	28864.67	10935.70	16786.36	25767.15	13049.94	17791.50	24255.84	7598.80	10574.82	14716.37

Sample: Saliva<br>Detector: FcγR3a	WT Spike 1			Delta Spike 1			BA1 Spike 1			BA2 Spike 1		
	Lower CI	Mean	Upper CI	Lower CI	Mean	Upper CI	Lower CI	Mean	Upper CI	Lower CI	Mean	Upper CI
Initial MFI:	81.46	177.83	388.22	20.32	50.12	123.59	16.98	16.98	16.98	10.23	10.23	10.23
Growth rate:	0.02	0.22	2.61	0.00	0.22	10.40	0.00	0.08	2.99	0.04	0.16	0.64
Activation time (days):	5.28	8.00	12.13	3.43	7.24	15.28	3.68	6.62	11.92	1.66	4.35	11.36
Peak time (days):	7.00	10.07	14.51	5.97	9.30	14.48	5.07	8.50	14.26	5.69	9.49	15.82
Maximum MFI:	695.15	1797.52	4647.99	216.73	663.87	2033.50	26.12	46.70	83.51	64.77	215.22	715.20

Sample: Saliva<br>Detector: FcγR3a	WT Spike Trimer			Delta Spike Trimer			BA1 Spike Trimer			BA2 Spike Trimer		
	Lower CI	Mean	Upper CI	Lower CI	Mean	Upper CI	Lower CI	Mean	Upper CI	Lower CI	Mean	Upper CI
Initial MFI:	190.44	457.09	1097.08	113.26	281.84	701.33	31.80	66.07	137.25	18.33	41.69	94.78
Growth rate:	0.00	0.20	3305836.17	0.00	0.14	8.31	0.03	0.18	1.07	0.03	0.15	0.77
Activation time (days):	0.99	7.17	51.91	4.16	7.61	13.95	3.91	6.05	9.37	4.03	6.89	11.79
Peak time (days):	2.78	8.58	26.55	5.11	9.97	19.46	5.55	8.76	13.83	7.14	10.70	16.02
Maximum MFI:	913.69	2515.28	6924.24	592.77	1823.82	5611.56	296.24	785.77	2084.23	180.93	535.25	1583.47

Supplementary Table 4: List of detection antibodies used.

Antibody:	Clone:	Company:	Catalogue Number:
Mouse anti-human IgG1, biotinylated	MTG1218	Mabtech	3851-14-250
Mouse anti-human IgG2, biotinylated	HP6200	Mabtech	3852-6-250
Mouse anti-human IgG3, biotinylated	MTG34	Mabtech	3853-6-250
Mouse anti-human IgG4, biotinylated	MTG42	Mabtech	3854-6-250
Mouse anti-human total IgA, biotinylated	MT20	Mabtech	3860-6-250
Mouse anti-human IgM, biotinylated	MT22	Mabtech	3880-6-250
Mouse anti-human total IgG, biotinylated	MT78/145	Mabtech	3850-6-250
Mouse anti-human total IgG-PE	JDC-10	Southern Biotech	9040-09
Mouse anti-human IgG1-PE	HP6001	Southern Biotech	9054-09
Mouse anti-human IgG2-PE	HP6002	Southern Biotech	9070-09
Mouse anti-human IgG3-PE	HP6050	Southern Biotech	9210-09
Mouse anti-human IgG4-PE	HP6025	Southern Biotech	9200-09
Mouse anti-human IgA1-PE	B3506B4	Southern Biotech	9130-09
Mouse anti-human IgA2-PE	A9604D2	Southern Biotech	9140-09
Mouse anti-human IgM	SA-DA4	Southern Biotech	9020-09
